# Amelotin: an enamel matrix protein that experienced distinct evolutionary histories in amphibians, sauropsids and mammals

**DOI:** 10.1186/s12862-015-0329-x

**Published:** 2015-03-14

**Authors:** Barbara Gasse, Ylenia Chiari, Jérémie Silvent, Tiphaine Davit-Béal, Jean-Yves Sire

**Affiliations:** Institut de Biologie Paris-Seine, Université Pierre et Marie Curie, Evolution Paris-Seine, Paris, UMR7138 France; Department of Biology, University of South Alabama, Mobile, AL 36688 USA; Department of Structural Biology, Weizmann Institute of Science, Rehovot, 76100 Israel

**Keywords:** Enamel, Amelogenesis, Tetrapods, Bioinformatics, Gene expression, Evolution

## Abstract

**Background:**

Amelotin (AMTN) is an ameloblast-secreted protein that belongs to the secretory calcium-binding phosphoprotein (SCPP) family, which originated in early vertebrates. In rodents, AMTN is expressed during the maturation stage of amelogenesis only. This expression pattern strongly differs from the spatiotemporal expression of other ameloblast-secreted SCPPs, such as the enamel matrix proteins (EMPs). Furthermore, *AMTN* was characterized in rodents only. In this study, we applied various approaches, including *in silico* screening of databases, PCRs and transcriptome sequencing to characterize *AMTN* sequences in sauropsids and amphibians, and compared them to available mammalian and coelacanth sequences.

**Results:**

We showed that (i) AMTN is tooth (enamel) specific and underwent pseudogenization in toothless turtles and birds, and (ii) the *AMTN* structure changed during tetrapod evolution. To infer AMTN function, we studied spatiotemporal expression of *AMTN* during amelogenesis in a salamander and a lizard, and compared the results with available expression data from mouse. We found that *AMTN* is expressed throughout amelogenesis in non-mammalian tetrapods, in contrast to its expression limited to enamel maturation in rodents.

**Conclusions:**

Taken together our findings suggest that AMTN was primarily an EMP. Its functions were conserved in amphibians and sauropsids while a change occurred early in the mammalian lineage, modifying its expression pattern during amelogenesis and its gene structure. These changes likely led to a partial loss of AMTN function and could have a link with the emergence of prismatic enamel in mammals.

**Electronic supplementary material:**

The online version of this article (doi:10.1186/s12862-015-0329-x) contains supplementary material, which is available to authorized users.

## Background

In tetrapods, the forming enamel matrix is mainly composed of three enamel matrix proteins (EMPs) secreted by ameloblasts: amelogenin (AMEL), ameloblastin (AMBN), and enamelin (ENAM). Three ameloblast-secreted proteins are further known: odontogenic ameloblast-associated (ODAM), amelotin (AMTN) and secretory calcium-binding phosphoprotein-proline-glutamine-rich 1 (SCPP-PQ1) [1-5]. These three proteins play a role during enamel maturation, but their precise function is still debated. Similar to EMPs, AMTN is a proline/glutamine (P/Q) rich protein that belongs to the secretory calcium-binding phosphoprotein (SCPP) family, which comprises proteins involved in mineralization of bone and tooth (dentin and enamel) tissues. In mammals only, this family also includes saliva and milk proteins [6-8]. The SCPP family is originally related to the SPARC family that evolved through whole-genome duplications and gave rise to SPARC and SPARC-L1 in vertebrates [9-11]. Further duplications of SPARC-L1 gave rise to the first members of bone, dentin and enamel SCPPs. The SCPP family expanded rapidly through tandem duplications as illustrated by the presence of ten SCPP genes (including *AMTN*, *AMEL*, *ENAM* and *AMBN*) in the coelacanth genome [12]. *AMTN* and the three EMPs were, therefore, present in the ancestral sarcopterygian genome 450 million years ago (Mya) [13] and probably earlier. However, so far, even if a number of SCPPs were identified in the actinopterygian lineage (in teleost fishes), only two of them, SPP1 and ODAM, were recognized as having an ortholog in sarcopterygians [14].

In mammals, SCPP genes, with the exception of AMEL, are organized in two clusters (P/Q rich and acidic SCPPs) located on the same chromosome. In sauropsids (birds and reptiles), these clusters reside on two different chromosomes [15]. In all genomes explored so far, the AMTN gene is always located upstream *AMBN* and *ENAM* [11,16,17]. Because the EMPs derive from duplication events, AMEL, AMBN and ENAM are phylogenetically related [18]. The encoding EMP genes have been identified in the genome of many tetrapods, amphibians, sauropsids and mammals [15,19,20], and recently in the coelacanth genome [12]. Although *AMTN* is known in all mammalian lineages [17], so far it was found only in one lizard [14] and in one coelacanth [12] genome. AMTN is therefore poorly known in non-mammalian tetrapods.

In mammals, *AMTN* is composed of nine exons, which are all encoding the protein except for the first one. As for all SCPP genes, the signal peptide is encoded by exon 2. The mature protein is encoded by the 3′ end of exon 2 and the following exons [1]. The translated protein is rich in proline, leucine, glutamine and threonine (52% of total by weight), and contains a conserved casein kinase 2 (CK2) phosphorylation site in the region encoded by exon 7 (SxxE/D, where x represents any amino acid) [3]. Two other transcripts were identified in rats. These splice variants lack either exon 7 or exons 3 to 7 [3].

A recent evolutionary analysis of AMTN in mammals has pointed to several amino acids unchanged for over 200 million years (Ma), indicating important functional sites [17]. Furthermore, AMTN is also essential for enamel biology as demonstrated by the presence of pseudogenes in enamel-less mammals e.g., sloth and armadillo [17]. However, in contrast to *AMEL, AMBN* and *ENAM,* in humans no *AMTN* mutation has been identified yet as being responsible for a genetic disease (*i.e*. amelogenesis imperfecta).

Studies in rodents have shown that AMTN is a secreted protein [1,3] that is post-translationally modified [3,21], probably by O-glycosylation [22]. In addition, AMTN interacts with itself and with ODAM, but not with AMBN, ENAM or AMEL [23]. *AMTN* is expressed by maturation-stage ameloblasts in mouse incisors [1]. The protein is specifically localized to a basal lamina-like layer, between the ameloblasts and the enamel mineral surface in rat and mouse incisors [3,21,24]. In contrast, Gao et al. [25] reports AMTN expression in the bulk enamel matrix of incisors and molars during early stages of mouse amelogenesis, athough these resuts are thought to be artifactual [21].

The function of AMTN remains largely unknown and studies of its expression during amelogenesis in mice and rats led to various hypotheses, e.g. as being involved either in enamel matrix proteolysis [1,25], or in adhesion of ameloblasts to the enamel surface [3], or in the establishment of the final prismless layer at the enamel surface [21]. *In vitro* studies revealed that recombinant AMTN did not mediate attachment of any cell types [21], refuting any role in ameloblast adhesion. Transgenic mice that overexpressed *AMTN* under *AMEL* promoter (*AMEL* is expressed early during amelogenesis) showed a disorganized enamel, a feature consistent with a role of AMTN in disrupting the regular arrangement of hydroxyapatite prisms [26]. Moreover, a recent study has shown that a recombinant human AMTN promotes hydroxyapatite precipitation and that the SSEEL motif in its phosphorylated form is necessary for the mineralizing property. The authors suggest that AMTN plays a role in the mineralization of the compact, nonprismatic, superficial enamel layer during maturation [27].

Despite its importance in enamel maturation in mammals, very little is known on AMTN evolution (origin, relationships and evolutionary histories) and function in tetrapods. To address this issue, we (i) identified *AMTN* sequences in two amphibians and eight sauropsids, (ii) compared the gene structure and the amino acid composition of the protein with those from mammals and coelacanth, (iii) compared the spatiotemporal expression of *AMTN* during amelogenesis in a lizard and a salamander with the expression pattern described in rodents, and (iv) analyzed the differences in the light of tetrapod evolution.

## Methods

### Amelotin sequences from publically available databases

*AMTN* sequences were obtained from databases (Ensembl [28] and NCBI [29]). See Additional file 1 for detailed information.

(i) Sequences of six representative mammalian lineages were used for comparison with non-mammalian *AMTN*: the published sequences from *Homo sapiens* (human, Primates) and *Mus musculus* (house mouse, Rodentia), and four sequences used in our previous study [17]: *Bos taurus* (cow, Laurasiatheria), *Loxodonta africana* (African elephant, Afrotheria), *Monodelphis domestica* (gray short-tail opossum, Marsupiala), and *Ornithorhynchus anatinus* (platypus, Monotremata).

(ii) Computer-predicted sequence of the lizard *Anolis carolinensis* (green anole, Squamata) was used as template to find *AMTN* with BLAST (Basic Local Alignment Search Tool) in three additional sauropsid genomes available in the Whole Genome Shotgun (WGS) database: *Alligator mississippiensis* (American alligator, Crocodilia) and two snakes, *Ophiophagus hannah* (king cobra, Squamata) and *Python molurus* (Indian python, Squamata).

(iii) No hit was obtained when blasting the following genomes with sauropsid and mammalian *AMTN* sequences: the bird *Anas platyrhynchos* (mallard duck, Anseriformes)*,* the turtle *Chelonia mydas* (green sea turtle, Testudines), the anuran *Xenopus (Silurana) tropicalis* (clawed frog, Amphibia) and the African coelacanth *Latimeria chalumnae* (Coelacanthiformes). However, as *AMTN* is located immediately upstream *AMBN* in mammalian and reptilian genomes, we used a synteny-based approach. We extracted 100 kb of genomic DNA (gDNA) upstream *AMBN* in these four genomes and explored this region with UniDPlot [30], a software designed to screen DNA regions showing weak sequence similarity [31]. The search was performed using UniDPlot with mammalian and sauropsid *AMTN* sequences and provided a predicted *AMTN* sequence in these four species.

*AMTN* sequences obtained either from cDNA sequencing or from sequenced genomes available in public databases can be found in Additional file 2 and at [32].

### RNA extraction and PCR amplification of Amelotin transcripts

The lower jaw of a juvenile specimen of the following species was used for RNA extraction: *X. tropicalis*, *A. carolinensis, Python regius* (royal python, Squamata) and *Takydromus sexlineatus* (long-tailed lizard, Squamata). The primers were designed using Primer3 v.0.4.0 [33,34] taking into account the *AMTN* sequences of *X. tropicalis*, *A. carolinensis* and *P. molurus* obtained *in silico* (Additional file 3).

Immediately after dissection, the jaws were immersed in RNAlater (Qiagen). Total RNAs were extracted and purified (RNeasy Midi Kit; Qiagen, France), and converted into cDNA (RevertAid™ H Minus First Strand cDNA Synthesis Kit; Fermentas, France) using an oligo(dT)18 primer. *AMTN* transcripts were recovered by PCR amplification using GoTaq DNA polymerase (Promega, France), as previously described [15]. Sequencing was performed by GATC Biotech.

### Jaw transcriptome

#### Caiman crocodilus, 454 sequencing

The lower jaw - *i.e.* mainly containing teeth, bone, cartilage, skin, muscle, and other tissues - was dissected from a 2 year-old *C. crocodilus*, cut into small pieces of about 5 mm-long each and flash frozen in liquid nitrogen. Tissues were finely ground in liquid nitrogen with a mortar and pestle and extracted following the protocol previously described [35]. Total RNA quantity and quality was checked by NanoDrop spectrophotometer (Thermoscientific, Wilmington, DE, USA) and by a capillary electrophoresis in RNA 6000 Nano Lab-Chip (Agilent-Agilent Bioanalyzer). Four RNA extractions for a total of 17 μg were used for transcriptome sequencing. 454 sequencing (half a run) was commissioned to GATC Biotech (Konstanz, Germany).

#### Other species, Illumina sequencing

*P. waltl* (6 month-old), *T. mauritanica* (adult). The lower jaw was dissected, immediately immersed in RNA later (Qiagen), cut into small pieces, and frozen in liquid nitrogen. Samples were transferred into homogenization tubes containing 2.8 mm ceramic beads and 2 mL of RLT solution (Qiagen). Tissues were disrupted with the Minilys homogenizer (Ozyme), three times for 15 sec at 5,000 rpm. Total RNA was extracted following the protocol of RNeasy Fibrous tissue mini kit (Qiagen). The quantity and quality of RNA were analyzed by Experion RNA StdSens chip (Bio-Rad) and using an Experion Automated Electrophoresis system (Bio-Rad). Illumina sequencing [one run, 50 base pairs (bp) paired end] was commissioned to GATC Biotech.

### Transcriptome screening

The sequenced jaw transcriptomes (see below “Jaw transcriptome sequencing”) of the caudate amphibian, *Pleurodeles waltl* (sharp-ribbed salamander, Amphibia), and two sauropsids, *Caiman crocodilus* (spectacled caiman, Crocodilia) and *Tarentola mauritanica* (common wall gecko, Squamata) were assembled at ISEM-Montpellier 2 (France) and screened for *AMTN* transcripts with BLAST using the Montpellier Bioinformatics Biodiversity (MBB) platform [36].

### Alignment, signal peptide and post-translational sites

The protein-coding regions of *AMTN* were translated into amino acid sequences and aligned to mammalian AMTN using Se-Al v2.0a11 [37]. The putative signal peptides were analyzed using SignalP 4.1 [38,39] and putative remarkable sites were identified using Prosite database [40,41].

### Histological analyses

One adult *P. waltl,* and several young adults *A. carolinensis* (3 month- to 2 year-old) were used. Immediately after dissection, the lower jaw quadrants were fixed in a mixture of glutaraldehyde (1.5%) and paraformaldehyde (1.5%) in PBS for 2 h, at room temperature. They were demineralized for three weeks, under gentle agitation at 4°C, in the same fixative to which 5% EDTA was added. The solution was changed every two days. After washing overnight in PBS, tissues were post-fixed for 2 h in 1% osmium tetroxide, rinsed in PBS, dehydrated through a graded series of ethanol then immersed in propylene oxide prior embedding in Epon 812 (EMS). 2 μm-thick sections were obtained using a Reichert OMU-3 ultramicrotome, deposited on a glass slide, stained with toluidine blue, and photographed with an Olympus BX61 microscope equipped with a QImaging camera using Image Pro Plus software (Media Cybernetics, Bethesda, MD).

### *In situ* hybridization

#### cDNA cloning and RNA probe synthesis

*AMTN* transcript fragments were amplified from *A. carolinensis* (556 base pairs, bp) and *P. waltl* (703 bp) cDNA, using primers designed from the gDNA and transcriptome sequences, respectively (Additional file 3). Once each cDNA fragment of interest was recovered (QIAquick Gel Extraction Kit; Qiagen, France), 4 μl of the purified cDNA were inserted into a pCRII-TOPO plasmid vector containing T7 and SP6 promoters for *in vitro* RNA transcription (TOPO-TA cloning kit; Invitrogen, France), and transformed into competent *E. coli* TOP10F’ bacteria. Colonies containing the vector and the insert were selected for plasmid purification (QIAprep Spin MiniPrep Kit; Qiagen, France). Purified plasmids were linearized by PCR using M13 forward and reverse primers and the sense and antisense RNA probes were synthesized using SP6 and T7 RNA polymerases (Riboprobe Combination System SP6/T7; Promega, France) in the presence of digoxigenin-UTP (Roche, France) and purified (ProbeQuant G-50 micro columns; GE Healthcare, France).

#### *Tissue processing and* in situ *hybridization*

The jaws were dissected in quadrants and fixed overnight, at 4°C, in Formoy (30% formaldehyde 37%, 10% acetic acid and 60% ethanol) solution. Tissues were demineralized in 10% acetic acid for 20 to 30 days at room temperature and under gentle agitation. The solution was changed every two days. Samples were then dehydrated through an increasing series of ethanol, shortly immersed in toluene and embedded in Paraplast (Sigma, France). The sections (8 μm-thick) were obtained with a Leica RM2245 microtome and deposited on Superfrost PLUS slides (Fisher Scientific, France).

The sections were dewaxed in toluene, rehydrated through a decreasing series of ethanol, then in PBS, treated with proteinase K (0.6 μg/ml) for 5 min at 37°C, rinsed in PBS, post-fixed for 30 min in 4% paraformaldehyde, rinsed again in PBS and then in 2× SSC. The slides were incubated overnight, at 65°C, with the digoxigenin-labeled antisense probe (0.25 ng/μl) in the hybridization buffer (50% formamide, 10% dextran sulfate, 1× salt, 1× Denhardt, yeast tRNA 1 mg/ml). The following day, the slides were washed three times, at 65°C, in the washing buffer (50% formamide, 1× SSC, 0.1% Tween 20), and rinsed, at room temperature, in the Maleic Acid Buffer Tween (MABT), pH 7.5. Non-specific binding sites were blocked for 2 h in a blocking solution (2% blocking reagent, 20% goat serum in MABT). Then, the slides were incubated overnight with the anti-digoxigenin antibody coupled to alkaline phosphatase (final concentration 750 mU/ml) in the blocking solution. After four baths of MABT, the slides were rinsed in NTM pH 9.5 (NaCl, TrisHCl, MgCl_2_). The digoxigenin-labeled probes were revealed at 37°C using NBT/BCIP (nitro blue tetrazolium chloride/5-bromo-4-chloro-3-indodyl phosphate). The slides were mounted in Aquatex mounting medium (Merck, France), and photographed (Olympus BX61 microscope).

### Ethics statement

All animal experiments conformed to the directives of the European parliament and of the council of 22 September 2010 on the protection of animals used for scientific purposes (Directive 2010/63/EU) and the French Rural Code (Article R214-87 to R214-137, Decree n° 2013–118 of 1st February 2013).

## Results

### Amelotin gene structure in sarcopterygians

A total of 17 *AMTN* sequences of sarcopterygian species were obtained using *in silico* screening of public databases, RT-PCR on jaw cDNA, and screening of assembled jaw transcriptomes: six species representative of mammalian lineages (*Homo sapiens, Mus musculus, Bos taurus*, *Loxodonta africana, Monodelphis domestica* and *Ornithorhynchus anatinus*), eight sauropsids (two crocodiles: *Alligator mississippiensis* and *Caiman crocodilus*; three lizards: *Anolis carolinensis - AMTN* referred to as [GenBank gene ID: LOC100554538] -, *Tarentola mauritanica* and *Takydromus sexlineatus*; three snakes: *Ophiophagus hannah, Python molurus* and *P. regius*), two amphibians (one frog: *Xenopus tropicalis*; one salamander: *Pleurodeles waltl*), and one coelacanth (*Latimeria chalumnae*). Genbank accession numbers of *AMTN* sequences obtained from cDNA are indicated in Additional file 1. Most sequences included either complete or partial 5′ and 3′ untranslated regions (UTRs). No alternative splicing of *AMTN* was detected in the PCR products obtained from the cDNA of all non-mammalian species used in this study. In the gDNA sequences analyzed in GenBank, *AMTN* was always found upstream *AMBN*, from 5 kb in *X. tropicalis* to 73 kb in *B. taurus*.

The exon-intron boundaries of nine non-mammalian *AMTN* sequences were inferred either from the comparison of cDNA and gDNA sequences obtained for the same species (*A. carolinensis* and *X. tropicalis*) or from sequence comparison of related species (*P. molurus/P. regius/O. hannah, A. mississippiensis/C. crocodilus,* and *T. sexlineatus/T. mauritanica/A. carolinensis*). The *AMTN* sequences in *P. molurus* and *P. regius,* on the one hand, and in *A. mississippiensis* and *C. crocodilus,* on the other hand, were nearly identical (98.8% and 97.3% nucleotide identity, respectively). For *H. sapiens, M. musculus, B. taurus, L. africana, M. domestica* and *O. anatinus* the exons were previously identified [17]. For *P. waltl AMTN* (cDNA data only) the exon-intron boundaries were estimated from the alignment of all *AMTN* sequences. However, the limit between exon 4 and exon 5 could not be clearly defined because of the high variability of this region when compared to the homologous region in other *AMTNs*. For *L. chalumnae* (gDNA data only), the exons were identified with UniDPlot then aligned to other *AMTN* sequences.

**Figure 1:**
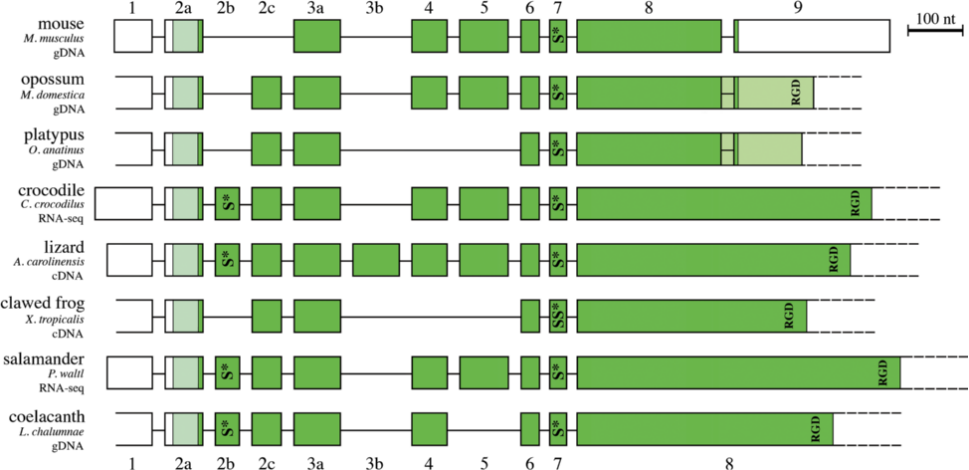
**Amelotin structure in the main sarcopterygian lineages.** The origin of the data is indicated below the species names. cDNA means that the sequences were obtained from PCR sequencing and RNA-seq means that the sequences result from transcriptome sequencing. The exon-intron boundaries were defined using comparison of cDNA and gDNA sequences (mouse, crocodile, lizard and clawed frog), or only cDNA (salamander), or only gDNA (opossum, platypus and coelacanth) sequences. The lizard gene structure is also applicable to snakes (*O. hannah* and *P. regius*). Two alternative ends of the opossum and platypus *AMTN* are proposed (dark and light green) depending on whether or not intraexonic splicing occurs in exon 8 (see text). A total of 12 exons (blocks) were identified. Coding regions in dark green; signal peptide in light green; non-coding regions in white. Introns (lines) are not at scale. S*: putatively phosphorylated serine.

**Figure 2:**
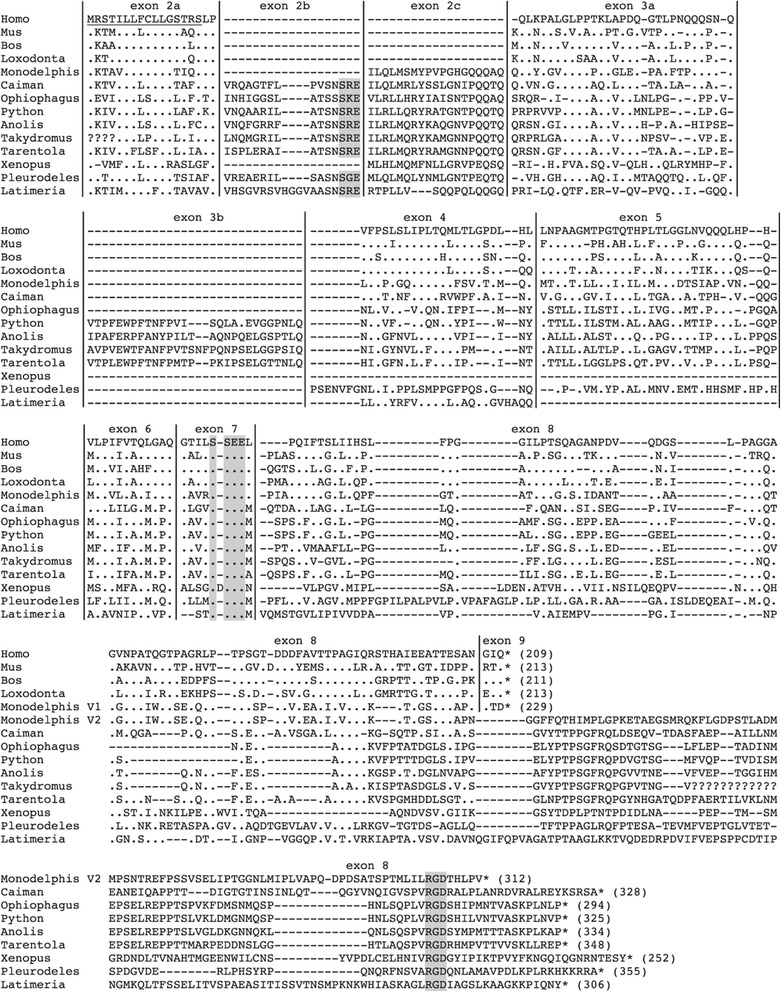
**Amino acid alignment of the Amelotin sequences used in our study.** The sequences are ordered according to species relationships (see Additional file 1 for names and references). Non-mammalian tetrapod sequences are aligned against the human sequence and four AMTN sequences representative of the main mammalian lineages. The coelacanth (*Latimeria*) sequence is aligned (only gDNA sequence) at the bottom. Two alternative ends of the opossum AMTN (*Monodelphis* V1 and V2) are proposed depending on whether or not intraexonic splicing occurs in exon 8 (see text). The specific length of each sequence is indicated in brackets at its end. Signal peptide underlined. SxE and SxxE motifs (encoded by exon 2b and exon 7, respectively) and RGD motif (encoded by the end of exon 8) highlighted in grey. Vertical lines: exon limits; (.): residue identical to human AMTN residue; (−): indel; (?): unknown amino acid; *: stop codon.

The *AMTN* structure resulting from these comparisons is represented in Figure 1 for the main sarcopterygian lineages (Placentalia, Marsupialia, Monotremata, Crocodilia, Squamata, Caudata, Anura and Actinistia). A total of 12 exons has been identified in the various sarcopterygian lineages, comprising the nine exons previously known in mammalian *AMTN* (exons 1–9), and three newly identified exons, *i.e.* two exons located between exons 2 and 3, and one exon inserted between exons 3 and 4. In order to conserve the current nomenclature of *AMTN* exons, we named 2b, 2c and 3b these three new exons, the former exons 2 and 3 becoming exons 2a and 3a (Figure 1). The largest number of exons identified in a single *AMTN* was eleven in squamates (lizards and snakes: exons 1, 2a, 2b, 2c, 3a, 3b, 4, 5, 6, 7 and 8; lack of exon 9), and the smallest was seven in *X. tropicalis* (lack of exons 2b, 3b, 4, 5 and 9). It is worth noting that the opossum *AMTN* (gDNA data only) may have either the same structure as in other mammals, *i.e*. the short exon 8 plus the exon 9 as suggested by Gasse et al. [17], or the structure in non-mammalian *AMTN*, *i.e.* only a large exon 8 as proposed by Kawasaki and Amemiya [12]. It is also possible that both *AMTN* sequences could be present in opossum as splice variants (Figures 1 and 2). Similar features could exist in platypus*,* for which also only *AMTN* gDNA is available (Figure 1).

The 5′ UTR of *AMTN* is composed of a variable number of nucleotides (nt), most in exon 1 plus 15 nt located at the beginning of exon 2a. In non-mammals, the sequence ends with exon 8 that is larger than in placental mammals and comprises the stop codon followed by the 3′UTR. The latter is also composed of a variable number of nucleotides. In placental mammals, the sequence ends with exon 9 that includes the termination codon and the 3′UTR. In non-placental mammals, platypus and opossum, both structures could occur (see below).

### Particular features of Amelotin in sarcopterygians

A single signal peptide (SP) encoded by exon 2a was detected in the N-terminal region of all AMTN obtained in this study (Figure 2). The SP cleavage site was predicted to occur between position 16 (either Ala, Cys, Gly or Ser, depending on the species) and 17 (Leu, Lys or Phe) with a probability > 0.9. In most AMTNs, the SP is composed of 16 (15 in *X. tropicalis*) amino acids (aa), and the first two residues of the mature protein are encoded by the last six nucleotides of exon 2a. Given the different *AMTN* structure in the various lineages, the length of the encoded protein varies from 252 aa in *X. tropicalis* to 355 aa in *P. waltl*.

Amino acid alignment indicated that the putative CK2 phosphorylation site (SxxE/D) previously identified in mammals in the region encoded by exon 7 is conserved in sarcopterygian AMTN (SseE) (Figure 2). In addition, in non-mammals a conserved SxE motif (putative phosphorylation site) was found in the region encoded by exon 2b, with the exception of *X. tropicalis AMTN* that lacks this exon. In the latter species, however, AMTN possesses a SxxD (SgsD: aa 78–81) and a SxE (SeE: aa 82–84) phosphorylation motifs adjacent one another in the region encoded by exon 7. A conserved Arg-Gly-Asp (RGD) motif (putative integrin-binding site) was found in the C-ter region encoded by exon 8 in non-mammals but it is missing in mammals, with the possible exception of marsupials (see below and Figure 2).

In addition to these features, in sauropsid *AMTN*, exon 2c and exon 3a encode the conserved motifs “PQQTQ” and “GLPPA”, respectively; also, the motif “KLVPD” encoded by exon 3a is well conserved in squamates. In the latter no particular motif is found in the AMTN region encoded by exon 3b (only found in squamates) while a conserved motif “PQQL” is encoded by exon 5. Also, exon 6 encodes 12 amino acids that are well-conserved in all species studied. No particular function is known for these conserved motifs.

### Intraexonic splicing in mammalian Amelotin exon 8

**Figure 3:**
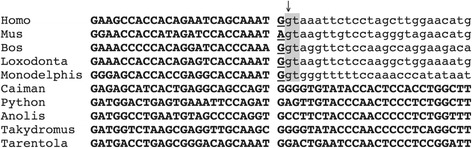
**Intraexonic splicing of Amelotin exon 8 in mammals.** The nucleotidic region of the *AMTN* exon 8 of five mammals and five sauropsids were aligned. Note the intraexonic splice donor site (arrow) that appeared in an ancestral mammal. A favorable environment for splicing (highlighted in grey) was created when the sequence was changed into Ggtg (then conserved in monotreme and marsupial lineages as shown here) and then into Ggta in an ancestral placental. Coding sequence in bold and upper case letters; new intron in lower case letters.

In non-mammals, *AMTN* exon 8 is in average 975 bp-long and encodes a variable region of the protein, the C-terminal region, which houses a well-conserved RGD motif (Figure 2). Exon 8 also encodes the stop codon, and contains the 3′UTR. In contrast, in placental mammals, exon 8 is shorter than in the other lineages. A large part of the 3′ region, including the region encoding the RGD motif, was lost and does not encode the termination codon, and is followed by intron 8 and exon 9. It has previously been suggested that the 3′ end of exon 8 has been spliced out in placental mammals [12]. Here, we provide an alignment of the 3′ extremity of mammalian exon 8 with the homologous intronic region in sauropsid sequences showing that nucleotide substitution in an ancestral mammalian exon 8 generated a favorable environment for an intraexonic splice donor site (Figure 3). This intraexonic splicing occurs within a codon, creating a phase 1 intron (*i.e.* the last nucleotide of exon 8 should be added to the two first nucleotides of the next exon to form a codon). A ninth exon possessing eight nucleotides followed by a stop codon and the 3′UTR was then recruited. In platypus and opossum, however, screening of the gDNA sequence provided two putative sequences depending on whether the intraexonic splicing occurs in exon 8 or not (Figure 2). In the former hypothesis the *AMTN* structure in the monotreme and marsupial lineages would be similar to that observed in placental mammals, *i.e.* a short exon 8 followed with exon 9 [17]. If the intraexonic splicing did not occur, an RGD motif would be encoded in the C-ter region of the opossum AMTN [12] but not in the platypus one (not shown). In opossum, the amino acids encoded by this exon 8 region can hardly be aligned to the homologous, non-mammalian sequences (Figure 2). In platypus alignment of this region is not possible (not shown).

### Amelotin in toothless sauropsids

**Figure 4:**
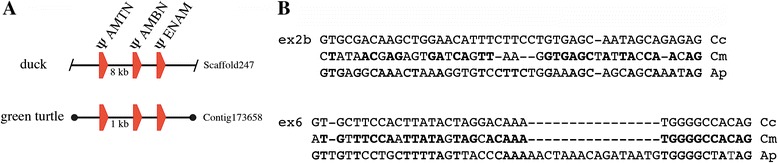
**Amelotin pseudogenization in two toothless sauropsids. A**. Location of pseudogeneized *AMTN* (*φ AMTN*) in the duck and green turtle genomes. In these two genomes, *φ AMTN* was found (synteny-based approach) upstream *φ AMBN* (ameloblastin) and *φ ENAM* (enamelin), all EMP genes being pseudogeneized in toothless sauropsids. **B**. Alignment of the nucleotide sequences of *φ AMTN* exons 2b and 6 of the green turtle *Chelonia mydas* (Cm) and the duck *Anas platyrhynchos* (Ap) with the corresponding coding exons of the crocodile *Caiman crocodilus* (Cc), their closest toothed relative. Note the few sequence variations in turtle *φ AMTN* exon 6. Conserved nucleotides in bold.

*AMTN* was searched in the sequenced genomes of the bird *Anas platyrhynchos* and of the turtle *Chelonia mydas* in order to check whether the gene is active or has been invalidated as a consequence of tooth loss in these two lineages. In the two species, our synteny-based approach allowed to identify *φ AMTN* in the gDNA region immediately upstream *φ AMBN,* as expected when taking into account the gene synteny observed in mammalian and sauropsid gDNA (Figure 4A)*.* The genomic distance between the two pseudogenes is 8 kb in *A. platyrhynchos* and 1 kb in *C. mydas.* These findings support that AMTN is only a tooth protein in the sauropsid lineage.

In *A. platyrhynchos,* most of the *φ AMTN* sequence is hardly recognizable in the non-coding gDNA region of scaffold247. Only two exons (2b and 6) could be identified within the 15 kb genomic region upstream *φ AMBN*. They display many nucleotide substitutions and indels (Figure 4B). In *C. mydas, AMTN* is also a pseudogene found in contig173658. Numerous mutations have accumulated in the sequence, but several exons (2a, 2b, 4, 5, 6, and 7) were identified using UniDPlot within the 10 kb upstream *φ AMBN*. They display less variations compared to *A. platyrhynchos AMTN* (Figure 4B).

### Searching for functional and invalidated exons in sarcopterygian gDNA sequences

Our results indicated the presence of a variable number of exons in sarcopterygian *AMTNs* (Figures 1 and 2). To verify whether exons were present but unidentified in some species, we screened target regions (intronic region between two exons) in sequenced genomes available in Genbank.

### Mammalian amelotin

In mammals, many *AMTN* sequences were previously obtained by blasting sequenced genomes, and the presence of nine exons was confirmed [17], but possibly eight in marsupials and seven in monotremes, depending on the intraexonic splicing in exon 8 described above. We looked for the putative presence of exons 2b, 2c and 3b in mammalian sequences by screening introns 2 and 3 of mammalian gDNA. We confirmed the presence of a putative exon 2c in *M. domestica AMTN* gDNA sequence (Figures 1 and 2)*,* then in the homologous genomic region of two other marsupials (Tasmanian devil: *Sarcophilus harrisii*; tammar wallaby: *Macropus eugenii*) and of a monotreme (platypus: *Ornithorhynchus anatinus*) (not shown). This exon 2c (57 bp) possessed high nucleotide identity with sauropsidian exon 2c and displayed correct putative splice sites, which suggests that exon 2c is functional in marsupial and monotreme *AMTN*. Exon 2c was not found in placental mammals but weak nucleotide similarity in the homologous genomic region of various mammalian species (not shown) suggests that exon 2c was present in an ancestral placental *AMTN,* then invalidated early, before the diversification of current placental lineages. In contrast, neither functional nor pseudoexons 2b and 3b were found in any mammalian genomes studied to date, which suggests that these two exons were either not present in the AMTN sequence of the last common ancestral mammal or invalidated early in the mammalian lineage, and no longer recognizable in gDNA.

### Sauropsid amelotin

In sauropsids, *AMTN* is composed of 11 exons in squamates (no exon 9) and of 10 exons in crocodiles (lack of exons 3b and 9) (Figures 1 and 2). The exon 9 found in all mammalian *AMTN* was identified neither in sauropsid gDNA nor in cDNA, all sequences ending with exon 8. Blasting the genomic region (*i.e*., 2 kb downstream exon 8) of sauropsids (*A. mississipiensis, A. carolinensis, P. molurus*, and *O. hannah*) using mammalian exon 9 sequence did not provide valuable hit. Exon 3b was identified in squamates only. We checked whether this exon was missed in *A. mississipiensis* gDNA, but no hit (even as pseudoexon) was obtained.

### Amphibian amelotin

In amphibians, *AMTN* was found to be composed either of 10 exons (absence of exons 3b and 9) in *P. waltl* (caudates) or of seven exons (absence of exons 2b, 3b, 4, 5 and 9) in *X. tropicalis* (anurans) (Figures 1 and 2). Exon 9 was identified neither in *P. waltl* nor in *X. tropicalis* cDNA, the two sequences ending with exon 8. Blasting the genomic region (*i.e*., 2 kb downstream exon 8) of *X. tropicalis* did not provide valuable hit. Similarly, exons 2b, 3b, 4 and 5 were not identified in the target genomic regions (introns 2 and 3) of *X. tropicalis* and no pseudoexons were found.

### Coelacanth amelotin

Exons 3b, 5 and 9 were not found in coelacanth *AMTN* (Figures 1 and 2). We did not identify any of them, even as pseudoexons, when blasting the target gDNA regions (introns 3 and 4, and 2 kb downstream exon 8).

### Amelotin expression during amelogenesis in lizard and salamander

**Figure 5:**
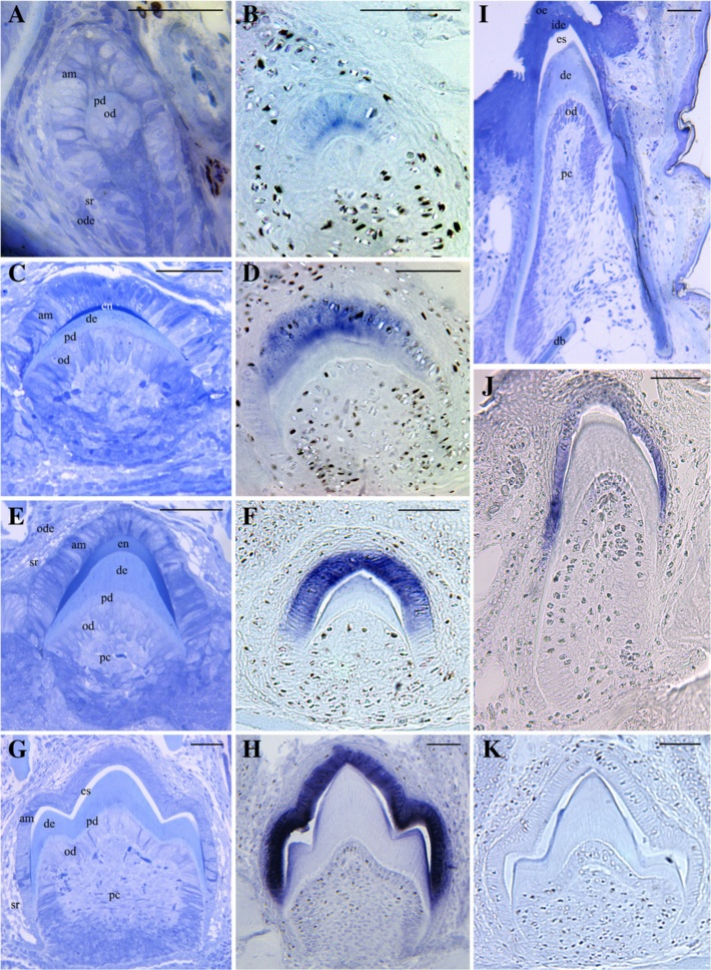
**Amelotin expression during amelogenesis in the iguanid lizard** ***Anolis carolinensis*** **.** Longitudinal **(A-H, K)** and transverse **(I, J)** sections of demineralized lower jaws (oral cavity on the top). In this lizard the dentition is heterodont: anterior teeth are monocuspid **(A-F, I, J)** and posterior teeth are tricuspid **(G, H, K)**. A, C, E, G, I: Histology, toluidine blue staining; **B**, **D**, **F**, **H**, **J**, **K**: *in situ* hybridization with *AMTN* antisense mRNA probe; K: Negative control with *AMTN* sense mRNA probe. **A, B**: Late cytodifferentiation. *AMTN* transcripts are weakly labelled in the ameloblasts lining the predentin matrix. **C**, **D**: Early deposition of enamel matrix. *AMTN* transcripts are detected in the well-polarized ameloblasts facing the enamel matrix (dark blue in C). **E**, **F**: Enamel deposition. The ameloblasts along the deposited enamel matrix are well labeled. **G**, **H**: Enamel maturation. *AMTN* mRNAs are strongly identified in the ameloblasts facing the enamel layer. The latter is well mineralized with the exception of the regions covering the minor mesial and distal cusps. **I**, **J**: *AMTN* expression is still detected during late maturation stage before the tooth attachment process begins. am: ameloblast, db: dentary bone; de: dentine; en: enamel; es: demineralized enamel space; ide: inner dental epithelium; od: odontoblasts; ode: outer dental epithelium; oe: oral epithelium; pc: pulp cavity; pd: predentin; sr: stellate reticulum. Scale bars = 50 μm.

**Figure 6:**
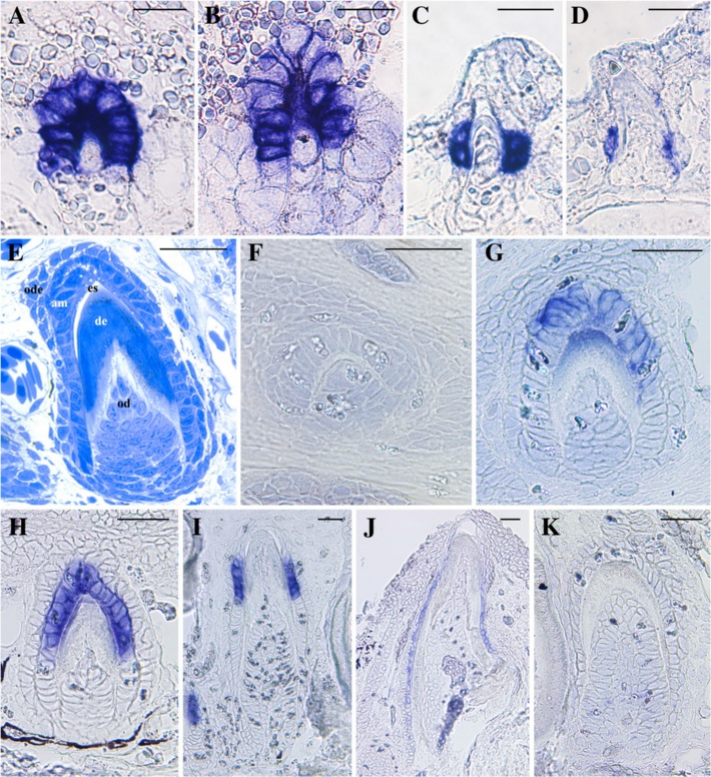
**Amelotin expression during amelogenesis in the salamander** ***Pleurodeles waltl.*** **A**-**D**: larval stages 34, 35, 36 and 38, respectively [42]; **E-K**: three year-old specimens. Transverse **(A-D, E, J)** and longitudinal **(F-I, K)** sections of demineralized jaws. **A-D, F-J**: *in situ* hybridization; **E**: histology; **K**: negative control. **A**: Enamel deposition on top of enameloid. The ameloblasts surrounding the enameloid cap express *AMTN*. **B**: Enamel maturation. Ameloblasts are strongly labelled along the crown base where enamel was recently deposited while labelling is weaker at its top where enamel is well mineralized. **C**: Late maturation stage. *AMTN* is no longer detected in the ameloblasts around the crown, but is identified in the cells at the surface of the dentin shaft, in a region lacking enamel. **D**: Tooth undergoing attachment process. Along the tooth base *AMTN* is still expressed in the cells of the inner dental epithelium. **E**: Differentiated tooth showing polarized ameloblasts. F: Early deposition of enamel matrix. *AMTN* transcripts are not detected, while thin layers of enamel and predentin matrix are observed. **G**: Enamel matrix deposition. *AMTN* mRNAs are clearly identified in the ameloblasts around the tooth top. **H**: Early enamel maturation. A strong *AMTN* expression is observed in the ameloblasts both facing the mineralized enamel and the recently deposited enamel matrix along the tooth shaft. **I**: Late enamel maturation. The ameloblasts are no longer labelled at the top of the tooth, where enamel is mature (empty space after demineralization), but are labelled in the cells facing the tooth matrix along the tooth shaft. **J**: Tooth in process of attachment. *AMTN* expression is visible in the inner dental epithelium cells facing the tooth matrix along the tooth base. am: ameloblasts; de: dentin; es: demineralized enamel space; od: odontoblasts; ode: outer dental epithelium. Scale bars: **A**-**D** = 25 μm; **E**-**K** = 50 μm.

Lizards and salamanders have polyphyodont dentitions - their teeth are continuously shed and replaced during the lifetime of the animal. Therefore, in a few, serially sectioned jaw quadrants of *Anolis carolinensis* and of *Pleurodeles waltl* we found various stages of tooth development, allowing the study of *AMTN* expression during various steps of amelogenesis in the two species (Figures 5 and 6).

### Anolis carolinensis

In the late cytodifferentiation stage, ameloblasts have differentiated from the inner dental epithelium of the enamel organ and they are facing mesenchymal cells of the dental papilla differentiated into odontoblasts. As soon as a thin layer of predentin was deposited between the ameloblasts and odontoblats, *AMTN* mRNAs were detected in the ameloblasts surrounding the forming teeth (Figure 5A, B). *AMTN* expression signal increased in intensity as enamel matrix was deposited and its mineralization started (Figure 5C-F). In tricuspid teeth, enamel matrix was deposited first on the central major cusp, then on the two adjacent minor cusps. *AMTN* expression weakened in the ameloblasts facing the major cusp at the time enamel maturation progresses, while *AMTN* transcripts were strongly labelled in ameloblasts surrounding the minor cusps (Figure 5G, H). *AMTN* expression was still detected in reduced ameloblasts during late maturation stage just before the tooth root attaches to the dental bone (Figure 5I, J). After eruption, when the tooth became functional, *AMTN* transcripts were no longer detected in the inner dental epithelial cells (not shown).

### Pleurodeles waltl

*AMTN* expression was monitored in larvae (from stage 34 onwards) and in adult specimens (Figure 6). In larval teeth dentin is covered first with enameloid then with enamel, while in juvenile and adult teeth, dentin is covered with enamel only. The onset of *AMTN* expression is detected earlier in larval than in adult teeth, but only during early deposition of enamel matrix (Figure 6A). In contrast, in adults labelling appears when a thick layer of dentin is already present (Figure 6F, G). During enamel maturation, *AMTN* expression is roughly similar in both larval and adult teeth: transcripts are identified in the ameloblasts around the tooth tip and in those facing the recently deposited enamel matrix along the crown base (Figure 6B, H). Once enamel is mature, *AMTN* expression is no longer detected in the ameloblasts around the upper region of the tooth but is detected in the cells of the inner dental epithelium facing the mid region of the tooth shaft (Figure 6C, I). Before tooth attachment to the dentary bone *AMTN* is still expressed in the inner dental epithelial cells facing the dentin base and the forming pedicel (Figure 6D, J). *AMTN* is no longer detected in the latter region after tooth eruption (not shown).

## Discussion

In this study, we combined RNA-seq data (transcriptomics), genomic data screening, isolation, characterization and expression of the AMTN gene to investigate the evolution of this SCPP in tetrapods. Our results suggest that AMTN was a component of the enamel matrix in the last common ancestor of tetrapods and that its structure changed and the function most likely changed in mammals, probably as a consequence of drastic events that occurred in the coding and/or regulatory region of the gene in the last common ancestor of mammals.

### The evolutionary history of Amelotin reveals important structural modifications in mammals

According to its genomic position, AMTN could be related to ENAM and AMBN because *AMTN* is located close to *AMBN* and *ENAM* in the same DNA region, and always upstream *AMBN* in the genome of sarcopterygian species annotated to date [12,18]. This vicinity suggests that *AMTN* and *AMBN* could be closely related SCPP genes, an hypothesis which is supported by the presence of remarkable motifs in the two proteins, in particular a SxxE motif encoded by a small exon in the middle region of the protein (exon 7 in AMTN, exon 12 in AMBN) [12]. Furthermore, the expression of *AMTN* and *AMBN* (but not that of *AMEL* or *ENAM*) is modified by the FAM20C kinase in mice [43]. Nevertheless, further data are needed in current representatives of basal actinopterygian lineages, *e.g.* polypterids and lepisosteids, and of chondrichthyans to better understand the evolutionary history of these EMPs in gnatosthomes.

**Figure 7:**
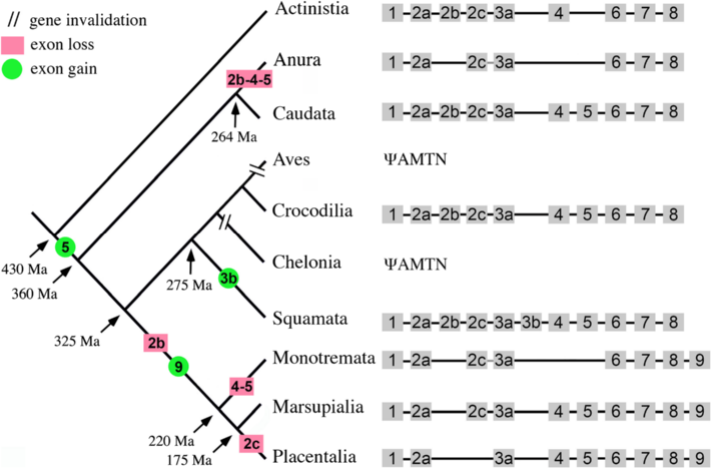
**Amelotin evolution in tetrapods.** Exons are shown as grey squares (not at scale). Species names used in this study are given in Additional file 1. *AMTN* was present in the last common ancestor of sarcopterygians and was either composed of 9 or 10 exons depending on the presence of exon 5 before or after the actinistian lineage (coelacanthiformes) separated from the other sarcopterygians. Distinct loss and gain of exons occurred in the various lineages explaining the current gene structure. *AMTN* was invalidated (φ *AMTN*) independently in chelonian (turtles) and aves (modern birds) after the capability to develop teeth was lost in both lineages. Two important events have been identified in the mammalian lineage: loss of exon 2b that encodes a phosphorylation site, and recruitment of exon 9 after exon 8 was shortened through intraexonic splicing, leading to the loss of the integrin-binding RGD motif. We can establish a parallel between these structural changes and the differences observed in *AMTN* expression during amelogenesis in mice and non-mammalian tetrapods (see text), suggesting important changes in the protein function. Estimation dates for lineage divergence and phylogenetic relationships are from [13,44-47].

The comparison of *AMTN* structure in sarcopterygian lineages suggests that the ancestral sequence was composed of either nine or 10 exons, depending on whether exon 5 was present in the ancestor and then lost in the coelacanth lineage (or not found in gDNA) or acquired in the lineage leading to tetrapods (Figure 7). To date *AMTN* transcripts, from which the *AMTN* structure could be accurately defined, are available neither in the coelacanth (only genomic sequence), nor in Dipnoi (lungfishes), nor in representatives of the basal actinopterygian lineages (polypterids, lepisosteids, acipenserids and amiids). Furthermore, when only gDNA is available coding exons could be missed, as illustrated by the discovery of the putative exon 2c in monotreme and marsupial *AMTN*, after this exon was identified in sauropsids. Indeed, this exon was not detected in opossum *AMTN* when using comparison with mammalian sequences that do not possess exon 2c [17] but was later found by Kawasaki and Amemiya [12] when using comparison to sauropsid sequences that include this exon.

Our results suggest that some coding exons present in the coelacanth *AMTN* and in most of the tetrapods analyzed here derive from those of an ancestral SCPP, from which *AMTN* and EMPs originated after duplication. Indeed, *AMTN* exons 2a and 2b are homologous to exons 2 and 3 (exon 4 and 5 in *ENAM*) of all SCPPs, respectively. In addition, AMTN shares several other characteristics with all EMPs, *i.e.* the signal peptide encoded by exon 2a, the conserved Ser-X-Glu motif (putative phosphorylation site) encoded by exon 2b, and the presence of a variable region encoded by a large exon, as previously observed by Kawasaki and Weiss [6].

The presence of *AMTN* exon 2b in salamander cDNA and its absence in clawed frog, for which gDNA and cDNA sequences are available, indicates that this exon has been lost either early in the anuran lineage (no *AMTN* sequences available in other frog lineages), or later in the lineage leading to Pipidae (Figure 7). Exon 2b also lacks in mammalian *AMTN*, a finding indicating that this exon was lost in an ancestral mammal, after mammalian and sauropsid lineages diverged. This would indicate that the phosphorylation site (SxE motif) encoded by exon 2b, the presence of which characterizes most SCPPs, is not required for the correct AMTN function during amelogenesis in clawed frog and mammals. This leads to the question of whether or not the phosphorylation site (SxxE motif) encoded by exon 7 compensates, in mammals, for the lack of that encoded by exon 2b.

Exon 2c was found in non-mammalian sarcopterygian *AMTN* and in non-placental mammals. The presence of this exon in representative species of the four sarcopterygian lineages (Actinistia, Amphibia, Sauropsida and Mammalia) indicates that its origin is to be found before the divergence of these lineages. On the other hand, its absence in placental mammals, suggests that exon 2c was probably lost in the common ancestor of placentals.

Exon 3b was found in squamate *AMTN* only, a finding which suggests that this exon was acquired either in the lepidosaurian lineage after its divergence from other sauropsid lineages or soon after Squamata diverged from the sphenodontian lineage (Figure 7).

Exon 4 is absent in the clawed frog *AMTN* sequence (gDNA and cDNA available), a finding which indicates that, as proposed for exon 2b this exon was lost either early in the anuran lineage or later, in the lineage leading to Pipidae. We did not find this exon in platypus *AMTN,* indicating that exon 4 was secondarily lost in this lineage. However, in absence of *AMTN* cDNA in this lineage, the lack of exon 4 could also result from a genome assembly artefact.

Exon 5 has not been found in coelacanth, clawed frog and platypus genomes, and is not present in clawed frog cDNA either. As discussed above we cannot know whether this exon was present in the sarcopterygian ancestor or was recruited later in a tetrapod ancestor. Parsimonious analysis suggests that exon 5 was secondarily lost in clawed frog (not found in clawed frog cDNA and gDNA sequence, but present in salamander *AMTN cDNA*) and in platypus, although the lack in the latter could result from a badly assembled genomic sequence.

Exon 7 encodes an SxxE motif (putative phosphorylation site) that is found in all AMTN sequences analyzed so far. When phosphorylated, this motif promotes hydroxyapatite precipitation *in vitro* [27]. Such motif is also found in two other EMPs, AMBN (encoded by exon 12) and ENAM (exon 9) [12]. These findings suggest that (i) the ancestral sarcopterygian *AMTN* exon 7 possessed this SxxE motif, (ii) the motif is functional (conserved during more than 430 Ma), and (iii) *AMTN* exon 7 is homologous to *AMBN* exon 12 and to *ENAM* exon 8, all of them preceding the large exons 8, 13 and 10, respectively. In clawed frog *AMTN*, the exon 7 not only encodes a SxxE/D motif, but also codes for a second phosphorylation site. Because the SxE motif encoded by exon 2b was lost in the pipid (or anuran) lineage, we suggest that the second SxE motif, encoded by exon 7, could compensate for the loss of the former. The presence of two phosphorylation sites, that could be located in different regions of the protein, was probably essential for AMTN to fulfill its function in non-mammalian sarcopterygians.

Exon 8 experienced two different evolutionary histories in sarcopterygians. In non-mammals, exon 8 is large and encodes a variable region of the protein, the C-terminal region of which includes a well-conserved Arg-Gly-Asp (RGD) integrin-binding motif that likely plays a crucial role for the protein function. RGD motifs are recognized by integrins and mediate the attachment of the cell to the extracellular matrix [48]. As for the last exon of all EMPs and of many SCPPs, exon 8 encodes also the termination codon and contains the 3′UTR. In contrast, in all placentalia lineages, *AMTN* exon 8 is shorter than in the other lineages and has lost a large part of the 3′ region, including the region encoding the RGD motif in non-mammals [12], and hence the loss of the adhesive function provided by this motif [48]. In addition, placentalia *AMTN* possesses an exon 9, which encodes the three last amino acids of the protein followed by the stop codon and the 3′UTR. Sequence comparison showed that a mutation in exon 8 generated an intraexonic splice site in an ancestral placentalia *AMTN*. This intraexonic splicing occurred within a codon, leading to the recruitment of exon 9 encoding an appropriate stop codon. It appears that encoding the stop codon could be the only role of exon 9 as several mammalian species lack this last exon because a mutation generated a stop codon at the extremity of exon 8 [17].

In monotremes and marsupials, the question of the efficient presence of the intraexonic splicing is posed. Indeed, the unavailability of cDNA data in both lineages has led to various interpretations. The first possibility we already proposed [17] is that intraexonic splicing occurred early in mammalian evolution, prior to current mammalian lineages differentiated. This finding is supported by (i) platypus and opossum *AMTNs* displaying a similar environment favorable for intraexonic splicing as observed in the homologous region of placental mammals, and (ii) exon 9 sequence, similar to that in placental mammals, present in both gDNA. The second possibility, suggested by Kawasaki and Amemiya [12], is that the intraexonic splicing in *AMTN* exon 8 did not occur in an ancestral marsupial but took place later in an ancestral placental. This finding is supported by the conservation of an RGD motif in the extremity of opossum AMTN, as in non-mammalian species. Such a long lasting conservation of RGD motif, although the rest of the sequence is less conserved, could indicate that this region is functional. A third interpretation could be that both AMTN variants are present in marsupials, the one with a short exon 8 and exon 9 (no RGD encoded), the other with the large exon 8 only (encoding an RGD). In platypus, the interpretation could be the same. However, if the intraexonic splicing does not occur and a large exon 8 transcribed, the latter does not encode an RGD. This suggests a different evolution of AMTN in this lineage. We previously showed in mammals that AMTN is enamel specific [17]. In platypus, enamel is no longer present in adults, meaning that the functional pressure on enamel proteins is relaxed [49]. This could have led to some changes in *AMTN* sequence, including mutations in the sequence encoding the RGD motif.

A RGD motif is present in all members of the acidic SCPPs (dentin and bone SCPPs) and is also found in the ENAM sequence of several mammalian species [50]. This suggests that the RGD motif of AMTN was probably inherited from an ancestral SCPP. Human and mouse recombinant AMTN do not mediate attachment of any cell types [21], consistent with the absence of RGD motif in mammalian AMTN.

Taken together our results suggest that in non-mammalian sarcopterygians AMTN may play an important role in ameloblast adhesion to the enamel matrix. This role in ameloblast adhesion was then probably lost, either in the last common mammalian ancestor or later in the last common ancestor of placentals, as a consequence of a mutation generating an intraexonic splice site in exon 8, suppressing the encoded RGD motif. Could the absence of this RGD motif explain the late expression of AMTN, *i.e.* from the maturation phase onwards, previously reported during murine amelogenesis [1,3,24]? The comparative study of *AMTN* expression in two selected tetrapods has answered partially this question.

### Drastic changes of Amelotin expression during amelogenesis occurred in early mammals

In lizard and salamander, tooth formation is roughly similar to that described in other vertebrates, but unlike mammals, ameloblasts do not exhibit Tomes’ processes, rod and interrod enamel structures are absent, and hence enamel is not prismatic [51-53]. Therefore important changes (Tomes’ processes, prismatic enamel) appeared in the mammalian lineage after it diverged from sauropsids, and before the divergence of marsupials, because the main ameloblast features and enamel structure are observed in current marsupial representatives, *e.g.* opossum [54]. These changes could be related to variation in spatial-temporal expression of the ameloblast-secreted proteins/genes during enamel matrix formation in mammals compared to non-mammalian tetrapods. In previous studies, we have shown that *AMEL* expression during amelogenesis was similar in all tetrapods [52,55]. No data are currently available in the literature for the expression of other ameloblast-secreted genes such as *AMBN*, *ENAM*, or *MMP20* in non-mammalian tetrapods.

Differences in the spatial-temporal expression of *AMTN* appear when comparing lizard or salamander amelogenesis to the one of rodents. In the incisors and molars of the latter, *AMTN* is predominantly expressed in maturation-stage ameloblasts [1]. In lizard, *AMTN* expression is detected in ameloblasts as soon as enamel matrix deposition has started, and is maintained from secretion to late maturation stage. *AMTN* is no longer expressed in the reduced ameloblasts after tooth eruption. In salamander larvae, *AMTN* expression is detected in early stages of amelogenesis while in adults, the onset of expression is delayed and appears slightly later than in the lizard, during enamel matrix deposition. Then, in both larvae and adult salamanders, *AMTN* expression is maintained in ameloblast until maturation stage. The disappearance of *AMTN* signal in ameloblasts occurs earlier than in lizard but it persists longer in the inner dental epithelium along the dentin cone. This late *AMTN* expression in the cells facing the region lacking enamel along the tooth base could be related to the formation of the amphibian pedicel [56,57]. Future studies should investigate this further. In erupted teeth of rats, a late expression of AMTN was detected in the internal basal lamina of the junctional epithelium [3,24], although no mRNA expression has been detected in the junctional epithelium of post-eruptive mouse molars [1,58]. In rodents, neither expression of the *AMTN* gene nor the protein was ever detected beyond the cemento-enamel junction in the epithelial cells along the tooth root. Nevertheless, AMTN was found in Hertwig’s epithelial root sheath cells entrapped in cementum [59]. In lizard and salamander, *AMTN* expression is detected earlier in secretory stage ameloblasts, and later in the cells of the inner dental epithelium along the tooth root. Immunolocalization of the protein should be performed to better understand the significance of the mRNA expression pattern.

*AMTN* being expressed during the various steps of amelogenesis in lizard and salamander, we concluded that (i) AMTN must be considered an enamel matrix protein (EMP), as are AMBN, ENAM and AMEL, and (ii) this status was the ancestral condition in non-mammalian tetrapods. When comparing lizard and salamander to mammals, one could hypothesize that the differences observed both in the timing of *AMTN* expression and in the gene structure could be responsible for the differences in enamel microstructure (non-prismatic versus prismatic, respectively) in these lineages. The absence of both Tomes’s process and of an organized rod/interrod enamel structure in lizard and salamander, on the one hand, and in mice overexpressing *AMTN* under *AMEL* promoter, on the other hand, support this hypothesis [26].

### An evolutionary scenario for Amelotin

The putative evolutionary scenario we propose to explain how the spatiotemporal *AMTN* expression and the gene structure changed in mammals could be visualized as the succession of several events that occurred early in mammalian evolution. One event probably occurred at the promoter level (mutation of the promoter, change in transcription factor, etc.) resulting in late *AMTN* expression during enamel mineralization. Two other, probably independent events resulted in the loss of exon 2b encoding the SxE motif and in the intraexonic splicing of exon 8 eliminating the RGD motif. The occurrence of these events (in any order) may have led to important changes in ameloblast structure (differentiation of Tomes’ processes) and in enamel organization (formation of prisms).

### Amelotin was tooth specific in early amniotes

In birds, pseudogenization of EMP genes is related to tooth loss [15,31,60]. In *A. platyrhynchos*, we found that *AMTN* is also a pseudogene. As observed for EMP genes, *AMTN* underwent pseudogenization after the capability to develop teeth was lost in the common ancestor of modern birds, 80–100 Mya [49,61]. Numerous mutations were accumulated (substitutions and indels), producing a *φ AMTN* sequence that is now hardly recognizable from the non-coding gDNA. In turtles, the capability to build teeth was lost in the last common ancestor of chelonians, 250 Mya [49]. In *C. mydas, AMTN* also underwent pseudogenization, producing a *φ AMTN,* in which a few exons could be identified. These findings indicate that in the ancestors of these two sauropsid species AMTN had no other functions than being involved in amelogenesis and is, therefore, an enamel specific protein in toothed sauropsids. In addition, it appears that, after the loss of function, mutations in this gene accumulated more rapidly in birds than in turtles although teeth were lost three folds earlier in turtle than in the bird ancestor [49]. This could be related to a slower evolution of chelonians during a long geological period (long generation time) compared to rapid evolution of neornithes (short generation time) [60,62].

In a previous study, we showed similar *AMTN* pseudogenization in two xenarthran mammals, armadillo and sloth, in which teeth are devoid of enamel cover [17]. These results therefore suggest that AMTN was tooth/enamel specific in the last common amniote ancestor, similarly to what was observed for other EMPs.

## Conclusions

This is the first comparative study of AMTN sequence in representatives of various mammalian and non-mammalian vertebrates. AMTN was present in the last common sarcopterygian ancestor more than 420 Mya. Various loss or gain of coding exons were identified in coelacanth, amphibians, reptiles and non-placental mammals, indicating different evolutionary pathways in sarcopterygian lineages. *In situ* hybridization of *AMTN* during tooth formation in lizard and salamander revealed that *AMTN* is expressed in ameloblasts during all stages of amelogenesis (with some differences in both species). These findings suggest that AMTN is an essential EMP for amelogenesis in these two species. The differences in spatiotemporal expression of *AMTN* described in murine *vs* lizard and salamander teeth would then suggest that in sauropsids and amphibians AMTN could have different functions than in mammals.

## Additional files

Additional file 1:
**Scientific and common names of the species used in our study, and sources of Amelotin sequences.** Last access to databases was on February 2014. The sequences obtained from sequenced genomes (names in bold) are available at [32]. Additional file 2:
**Amelotin sequences.** In bold: from cDNA sequences. ?: unknown residues. *: Stop codon. Additional file 3:
**Forward (F) and reverse (R) primers used to amplify Amelotin cDNA of various species and to build** ***AMTN*** **probes.** Target regions and annealing temperatures are indicated.

## Abbreviations

Aa: Amino acids
AMBN: Ameloblastin
AMEL: Amelogenin
AMTN: Amelotin
BLAST: Basic local alignment search tool
bp: base pair
CK2: Casein kinase 2
EMPs: Enamel matrix protein
ENAM: Enamelin
Ma: Million years
MABT: Maleic acid buffer tween
MBB: Montpellier bioinformatics biodiversity
Mya: Million years ago
NBT/BCIP: Nitro blue tetrazolium chloride/5-bromo-4-chloro-3-indodyl phosphate
Nt: Nucleotides
NTM: NaCL, TrisHCl, MgCl2
ODAM: Odontogenic ameloblast-associated
RGD: Arg-gly-asp
SCPP: Secretory calcium-binding phosphoprotein
UTRs: Untranslated regions
WGS: Whole genome shotgun
