# Supplementary material for: Amelotin: an enamel matrix protein that experienced distinct evolutionary histories in amphibians, sauropsids and mammals
Source: BMC Evol Biol. 2015 Mar 14;15:47. doi: 10.1186/s12862-015-0329-x (PMC4373244; doi:10.1186/s12862-015-0329-x)
Supplement: Additional file 1: — Scientific and common names of the species used in our study, and sources of Amelotin sequences. Last access to databases was on February 2014. The sequences obtained from sequenced genomes (names in bold) are available at [32]. [file 12862_2015_329_MOESM1_ESM.pdf]

**Genus Species**	**Common name**	**Family and Order**	**Sources**
Mammalia			
Bos taurus	Cow	Bovidae, Artiodactyla	XM_870725
Homo sapiens	Human	Hominidae, Primates	NM_212557
Loxodonta africana	African elephant	Elephantidae, Proboscidea	XM_003414063
Monodelphis domestica	Opossum	Didelphidae, Didelphimorphia	XM_007495514
Mus musculus	Mouse	Muridae, Rodentia	NM_027793.1
Ornithorhynchus anatinus	Platypus	Ornithorhynchidae, Monotremata	OANA5
Sauropsida			
Alligator mississippiensis	American alligator	Alligatoridae, Crocodilia	allMis0.2
Anolis carolinensis	Green anole	Iguanidae, Squamata	XM_008103694 / KM069435
Caiman crocodilus	Spectacled caiman	Alligatoridae, Crocodilia	KM069444
Chelonia mydas	Green sea turtle	Cheloniidae, Testudines	CheMyd_1.0
Anas platyrhynchos	Mallard duck	Anatidae, Anseriformes	BGI_duck_1.0
Ophiophagus hannah	King cobra	Elapidae, Squamata	SAMN02439592
Python molurus	Indian python	Pythonidae, Squamata	PytMol_1.0
Python regius	Royal python	Pythonidae, Squamata	KM069438
Takydromus sexlineatus	Long-tailed lizard	Lacertidae, Squamata	KM069441
Tarentola mauritanica	Common wall gecko	Gekkonidae, Squamata	KM069446
Amphibia			
Pleurodeles waltl	Sharp-ribbed salamander	Salamandridae, Caudata	KM069436
Xenopus (Silurana) tropicalis	Clawed frog	Pipidae, Anura	KM069437
Coelacanthid			
Latimeria chalumnae	Coelacanth	Coelacanthidae, Coelacanthiformes	LatCha1

Additional file 1.
