# Supplementary material for: Amelotin: an enamel matrix protein that experienced distinct evolutionary histories in amphibians, sauropsids and mammals
Source: BMC Evol Biol. 2015 Mar 14;15:47. doi: 10.1186/s12862-015-0329-x (PMC4373244; doi:10.1186/s12862-015-0329-x)
Supplement: Additional file 2: — Amelotin sequences. In bold: from cDNA sequences. ?: unknown residues. *: Stop codon. [file 12862_2015_329_MOESM2_ESM.pdf]

Additional file 2. Amelotin (AMTN) sequences. In bold: from cDNA sequences. ?: unknown residues. *: Stop codon.

MAMMALIA

Primates

>AMTN Homo sapiens [NM_212557.2]
MRSTILLFCLLGSTRSLPQLKPALGLPPTKLAPDQGTLPNQQQSNQVFPSLSLIPLTQMLTLGPDLHLLNPAAGMTPGTQTHPLTLGGLNVQQQLHPHVLPIFVTQLGA
QGTILSSEELPQIFTSLIIHSLFPGGILPTSQAGANPDVQDGSLPAGGAGVNPATQGTPAGRLPTPSGTDDDFAVTTPAGIQRSTHAIEEATTESANGIQ*
>AMTN Pan troglodytes
MRSTILLFCLLGSTRSLPQLKPALGLPPTKPAPDQETLPNQQQSNQVFPSLSLIPLTQMLTLGPDLHLLNPAAGMTPGTQTHPLTLGGLNVQQQLHPHVLPIFVTQLGA
QGTILSSEELPQIFTSLIIHSLFPGGILPTSQAGANPDVQDGSLPAGGAGVNPATQGTPAGRLPTPSGTDDDFAVTTPADIQRSTRAIEEATTESANGIQ*
>AMTN Pan paniscus
MRSTILLFCLLGSTRSLPQLKPALGLPPTKLAPDQGTLPNQQQSNQVFPSLSLIPLTQMLTLGPDLHLLNPAAGMTPGTQTHPLTLGGLNVQQQLHPHVLPIFVTQLGA
QGTILSSEELPQIFTSLIIHSLFPGGILPTSQAGANPDVQDGSLPAGGAGVNPATQGTPAGRLPTPSGTDDDFAVTTPADIQRSTRAIEEATTESANGIQ*
>AMTN Gorilla gorilla
MRSTILLFCLLGSTRSLPQLKPALGLPPTKLAPDQGTLPNQQQSNQVFPSLSLIPLTQMLTLGPDLHLLNPAAGMTPGTQTHPLTLGGLNVQQQLHPHMLPIFVTQLGA
QGTILSSEELPQIFTSLIIHSLFPGGILPTSQAGANPDVQDGSLPAGGAGVNPAIQGTPAGRLPTPSGTDDDSAVTTPAGIQRSTRAIEETTIESANGIQ*
>AMTN Pongo pygmaeus
MKTTILLFCLLGSTRSLPQLKPALGLPPTKRAPDQGTLPNQQQSNQVFPSLSLIPLTQMLTLGPDLHLLNPAAGMTPGTQTHPLTLGGLNVQQQLHPHMLPIFVTQLGA
QGTILSSEELPQIFTSLIIHSLFPGGILPTSQAGANPDVQDGSLPGGGAGVNPAIQGTPAGRLPTPSGTDDDFAVTTPAGIQRSTRAIEETTTESANGIQ*
>AMTN Pongo abelii
MKTTILLFCLLGSTRSLPQLKPALGLPPTKRAPDQGTLPNQQQSNQVFPSLSLIPLTQMLTLGPDLHLLNPAAGMTPGTQTHPLTLGGLNVQQQLHPHMLPIFVTQLGA
QGTILSSEELPQIFTSLIIHSLFPGGILPTSQAGANPDVQDGSLPGGGAGVNPAIQGTPAGRLPTPSGTDDDFAVTTPAGIQRNTRAIEETNTESANGIQ*
>AMTN Nomascus leucogenys
MKTTMLLFCLLGSTRSLPQLKPALGLPPTKLAPDQGTLPNQQQSNQVFPSLSLIPLTQMLTLGPDLHLLNPAAGMTPGTQTHPLTLGGLNIQQQLHPHILPIFVTQLGA
QGTILSSEELPQIFTSLIIHSLFPGGILPTSQAGANPDVQDGSLPAGGAGVNPAIQGTPAGHLPTPSGTDDDFAVTTPAGIQRNTHAIEETTTESANGIE*
>AMTN Macaca mulatta
MKTMILLFCLLGSTQSLPQLKPALGLPPTKLAPDQGTLPNQQQSNQVFPSLSLIPLTQMLTLGPDLHLLNPAAGMTPGTQAHPLTLGVLNLPQQLQPHMLPIFVTQLGA
QGTILSSEELPQIFTSLIIHSLFPGGILPTSQAGANPDVQDGSLPAGQAGVNPAIQGTPAGRLPTPSGTDDDFAVTTPAGIQRSTHATEETTTE*
>AMTN Macaca fascicularis
MKTMILLFCLLGSTQSLPQLKPALGLPPTKLAPDQGTLPNQQQSNQVFPSLSLIPLTQMLTLGPDLHLLNPAAGMTPGTQAHPLTLGVLNLPQQLQPHMLPIFVTQLGA
QGTILSSEELPQIFTSLIIHSLFPGGILPTSQAGANPDVQDGSLPAGQAGVNPAIQGTPAGRLPTPSGTDDDFAVTTPAGIQRSTHATEETTTE*
>AMTN_Nasalis larvatus
MKTMILLFCLLGSTQSLPQLKPALGLPPTKLAPDQGTLPNQQQSNQVFPSLSLIPLTQMLTLGPDLHLLNPAAGMTPGTQAHPLTLGVLNLQHQLQPHMLPIFVTQVGA
QGTILSSEELPQIFTSLIIHSLFPGGILPTSQAGVNPDVQDGSPPAGQAGVNPAIQGTPAGRLPTPSGTDDDFAVTTPAGIQRSTHATEETTTESPNGIQ*
>AMTN Papio anubis
MKTMLLLFCLLGSTQSLPQLKPALGLPPTKLAPDQGTLPNQQQSNQVFPSLSLIPLTQMLTLGPDLHLLNPAAGMTPGTQAHPLTLGVLNLQQQLQPHMLPIFVTQLGA
QGTILSSEELPQIFTSLIIHSLFPGGILPTSQAGANPDVQDGSLPAGQAGVNPAIQGTPAGRLPTPSGTDDDFAVTTPAGIQRSTHATEETTTE*
>AMTN Papio hamadryas
MKTMLLLFCLLGSTQSLPQLKPALGLPPTKLAPDQGTLPNQQQSNQVFPSLSLIPLTQMLTLGPDLHLLNPAAGMTPGTQAHPLTLGVLNLQQQLQPHMLPIFVTQLGA
QGTILSSEELPQIFTSLIIHSLFPGGILPTSQAGANPDVQDGSLPAGQAGVNPAIQGTPAGRLPTPSGTDDDFAVTTPAGIQRSTHATEETTTE*
>AMTN Callithrix jacchus
MKTTILLFCLLGSTQSLPQLKPALGLPPTKLALDQATLLNQQQSNQVFPSLSLIPLTQMLTLGQDLQLVKSCCRNDGWYPDPPIDPGRVECTTATATTCMLPIFVTHLG
AQGTILSSEELPLAPQIFTGLIIHSLLPGGILPTSQAGANPNVQDGRLPAGQAGVNSAIQGTPAGHLPIPSSTDDDFAMTTPAGIQRSTHAMEETTTESPNGIQ*
>AMTN Saimiri boliviensis
MKTTILLFCLLGSTQSLPQLKPALGLPPTKLAPDQATLLNQQQSNQVFPSLSLIPLTQVLTLGQDLQLLNPAAGMTPGAQTHPLTLGGLNVQQQLQPHMLPIFVTHLGA
QGTILSSEELPLAPQIFTGLIIHSLFPGSILPTSQAGANPNVQDGRLPAGQAGVNPAIQGTPAGHFPTPSSTDDDFAMTTPAGIQRSTHAVEETTTESSNGIQ*
>AMTN Microcebus murinus
MKTTILLFCLLGSTQSLPRHLNPALGLTPTKLAPDQATPLTQQQPNQVVPSLSVIPLTQMLTLGSDLHLLNPAVGMTPGLQAHPLPLGGLNVQQQLQPQVLPIFIAQLG
AQ?????????PVAPHFFTGLIIHPLLPGGILPPTQAGANPDGQGTSLPAGQAGVNPATHGTPEGHLPTPTDTDDEFGVTTPAGLRRGLHTTEETTTEPPNE???
>AMTN Daubentonia madagascariensis
MKTTILLFCLLGSTQSVPKHLNPALGLPPTKPAPDQATLLNQQQPNPVFPSLSLIPLTQMFALGSDLHLLNHAAGMTPGTQAHPLPLGGLNVQQQLQSQMLPIFVAQLG
AQGTILSSEELPVAPQIFTGLIIHPLFPGGILPTSQVGANPGGQGGSLPAGQAGVNPATHGTPEGHHPTPRDTDDEFGVTSPAGIHRGTHTTEETTTESPNGIQ*
>AMTN Tarsius syrichta
MKTTILLFCLLGSAQSLPKQLNPALGLPTKLAPDQVTLLNQQQPNQVFPLSLIPLTQMLTLGSDLQLLNPAAGMTPGAQTHPLTLGGLNVQQQLQPQMLPIFLAQLGAQ
GTILSSEELPVGSPFFTGLILHPLFPGGILPTSQAGTKPDVQDGSLPAGQTGANPAIQGTPAGLPTPSVTDDEFGVTTPASIQRGTHTVEETTTESPDGIQ*
>AMTN Otolemur garnettii
MKTMILLFCLLGPAQSLPKHLSPTLGLQPTKLAPDEATLPTQQQPNQVLPSISLIPLTQMLTLGSDLRLLSPAAGLAPGAQAHSPSLGGLNAQQLQPQMLPIFVAQLGA
QGTILSSEELPVAPQIFTGLIIHPLFPGGVLPASQARANLDVQGENLPAGTAEAHLTTPRDDDESGVTPPAGIRSGTSTGSDTTTELPNGIQ*

Dermoptera

>AMTN Galeopterus variegatus
MKTAILLFCLLGSTQSLPKQLNPALGLPPTKVALNQATPLNQQQPNQVFPSLSLIPLTQMLTLGSNLQLLHPTAGMTPGAQTLPLTLGALNVQHHLQPQMLPIFVAPLG
AQGTILSSEELPVAQQIFTGLIIHPLYPGDLLPSSQAGANPDVQARIPSAGQAGANPAFQRTLPTPRGTDDDFEVATPAGIQRGLHTTEETTTESPNGIQ*

Euarchontoglires

Scandentia

>AMTN Tupaia chinensis
MKTMILLFCLLGSTQSLPKQHNPASGLPPTKLAPDQAILLNQQQPKQVFPSLHLIPLTQLLTLGSDLQLLNPVAGMTPGAQTLPLALGRLNVQHQLQPQMLPIIVAQLG
AQGTVLSSEELPVAPQIFTGLIIHPLFPGGILSTSQTGANPEVHNGILPAGQAGVNPAIQGTPGGHLPTPSDTDDELDVATPAGILRGMHTTEETTTESPNGIQ*
>AMTN Tupaia belangeri
MKTMILLFCLLGSTQSLPKQHSPASGLPPTKLAPDQAILLNQQQPKQVFPSLHLIPLTQLLTLGSDLQLLNPVAGMTPGAQTLPLALGRLNVQHQLQPQMLPIIVAQLG
AQGTVLSSEELPVAPQIFTGLIIHPLFPGGILSTSQTGANPEVHNGILPAGQAGVNPAIQGTPGGHLPTPSDTDDELDVATPAGILRGMHTTEETTTESPNGIQ*

Rodentia

>AMTN Mus musculus [NM_027793]
MKTMILLLCLLGSAQSLPKQLNPASGVPATKPTPGQVTPLPQQQPNQVFPSISLIPLTQLLTLGSDLPLFNPAAGPHGAHTLPFTLGPLNGQQQLQPQMLPIIVAQLGA
QGALLSSEELPLASQIFTGLLIHPLFPGAIPPSGQAGTKPDVQNGVLPTRQAGAKAVNQGTTPGHVTTPGVTDDDDYEMSTPAGLRRATHTTEGTTIDPPNRTQ*
>AMTN Rattus norvegicus [NM_001044296]

MKTVVLLLCLLGSAQSLPRQLSPALGAPATKPTPGQVTPLTQQQPNQVFPSISLIPLTQLLTLGSDLPLFNPATMPHGTQTLPFTLGPLNGQQQLQPQMLPIIVAQLGA
QGALLSSEELPLASQIFTGLLIHPLFPGAIQPSGQTGAKPDVQNGALPTRQAGASPANQATTPGHTTPAVTDDDDYEMSTPAGLQRATHTTEGTTMDPPNRTK*
>AMTN Cricetulus griseus
MKTTILLFCLLGSTQSLPKQISPTLGLPPTKTADQVALLTQQQPNQVFPSISLIPLTQLLTLGSDLQLLNPATGLAHGAQALPFTLGPLNRQQQLQSQMLPIIVAQLGA
QGTLLSSEELPLASQIFTGLLIHPLFPGGILPSSQTGAKPDGQNGVLPTRQAGANPAVQGTTQGHVTTPGVTDDDDYETSTPAGIRRGTHTTEGTTIDTPNRTQ*
>AMTN Mesocricetus auratus
MKTTILLFCLLGSTQSLPKQISPALGLPATKTADQVTLLTQQQSNQVFPSISLIPLTQLLTLGSDLQL??????????????????????????????MLPIIVAQLGA
QGTLLSSEELPLASQIFTGLLIHPLLPGGILPSSQTGAKPDVQNGVLPTRQAGANPAVQGTTQGYITTPGVTDDDDYEMSTPAGIRRGTHTTEGTTIDTPNRTQ*
>AMTN Peromyscus maniculatus
MKTTILLFCLLGSTQSLPKQIRPTLGFPPTTSPDQGTRLTQEQANQVFPSISLIPLAQLLTLGSDLQLLNPATGPAHGTQTFPFPLGPLNRPQQLQPQMLPIIVAPLGA
QGALLSSEELPLASQIFTGLLIHHPLFPGGILPSSQTGAKPDAQTGVLPTRQAGASPAVQGTTQSQVTTPGVTDDDDYETSTPAGMRRGTHTTEGITLDPPNRTQ*
>AMTN Microtus ochrogaster
MKTTILLFCLLGSAQSLPKQINPALGLPPTKTPDQVALLSQQQPNQIFPSISLIPLAQLLTWNSDLQLLNPATGLAHGAQTLPFTLGPLSRSQQLQPQMLPIIVTQLGA
QGTFLSSEELPLASQIFTGLLIHPLFPGGILPSSQTGAKPDAQNGVLPTRQAGTSPATQGPTQGHVTTPAVTDDDDYEMSTAAGMRRGTHTTDGTTTDPPNRTHKDLLD
*
>AMTN Jaculus jaculus
MRTTILLLCLLGSTQSLPKQLNPASGLPPTKPAPDQVMLVKQQLPSQVLPSLSLIPLTQVLALGADLQLISPSTGVAPGTQTRPVSLGALNGQQQLQPQMLPIIVAQLG
AQGTLLSSEELPLASQVFTGLFLHPLFPGSILPTNQAGSNPDAQKGVLPAGQTGTIQGTTEGHIPVSTDDDFAMTTPAGLRKGVHTTGETTPESTSGIH*
>AMTN Dipodomys ordii
MKTAILLFCLLGSTQSLPKQPNPTLGYPPTKPTPEQVTLLNQQQSHQVFPSLSLIPLTHLFTLGSDLQLFNHATGIAPGTHTLPVTLGALNGQQQLPSQMLPIIVASLG
AQSTILSSEDSPLASQIFTGLLIHPLFPGNILPTSQTGPTPGAQDGVFPAGQAGVKPAIQGTTESHISTPSVTDDDFGGTTPAGIRRGMHTTVEETTAD*
>AMTN Cavia porcellus
MKTVILLLGLLGWAQSLPKQLNSALEVSPTKPAPDQVTPLSQQQPTQVFPSLSLISLTQLLTLASDLQLLTPASGTKPGAQTLQLSLGALQGQNQLQPQLLPIFVAQLG
APAAILSSEELPLAPQIFTGLLLNPLFPEGTLPTSQAGASPNIQEEAIPAGQTGVNTNMQVTTEGQLSTPGVTDDDFEVTTTLGIQKVTHTPEKTTTGSPN*
>AMTN Octodon degus
MKTAILLLCLLGSAQSLPKQLNSALEVSPTKPTPDQVTPQKQQQSSQVFPSLSLIPLTQLLTLGSDLQLLTPAPGATPAAHTLPLTLGTVNGQNQLQPQTLPIFVAQLG
AQAAILSSGELPLGPQIFTGLLLHPFFPGGILPISQAGANPDGQDGALPAGQTGANANIQATTVGQLSTSGVTDDDFEVTTPAGIQRATHTTKQTITGSPNRIH*
>AMTN Chinchilla laniger
aMKTAILLFCLLGSTQALPKQLNSALEVSPTKPAPDQVTPLSQQQPGQVFPSLSLIPLTHLLTLGSDLQLLSPAPGATPAAHTLPLPLGALNGQNQLQPQMLPIFVAQF
GAQAAILSSEELPLGPQIFTGLLLHPLFPGGILPTSQAGANPDGQDGALPTGQTGANANIQATTEGQLSTPGVTDDDFEVTTPAGIQKATHTTKETTTSSPNRIH*
>AMTN Heterocephalus glaber
MKTMILLFCLLGTTQSLPKQLNSALEVSPTKLAPDQVTPLTQQQPSQVFPSLNLIPLTQLLTLGSDLRLLTPAPGLAPGAQKLPLTLGALNGQNQQQPQMLPIFVAQIG
AQATILSSEELPLGSQIFTGLLLHPLFPGSILPTSQSGANLDAQNGALPGGQAGENANIQATPEGHLLTPGVTDDDFEATTPAGIQRATHTTKGITTGSPNGTQ*
>AMTN Fukomys damarensis
MKTLILLFCLLGSTRSLPKQLNSALEASPTKPAPDQVTTVTQQQPSQVFPSLSLIPLTQLLTLGSDLQLLTPAPGAAPGAQKLPLTLGALNGQNQLQQVLPIFVTQVGA
QATMLSSEELPLGSQFFTGLLLHTLFPGGILPTSQVGANPDAQDGALPVGQAGANANIQATPEGHLSTPGVTDDDFEATTPAGIQRATHTTKGITTGSPNGIH*
>AMTN Nannospalax galili
MKTTILLFCLLGSTQSLPKQLSPALGLPPTKPTPNQVTLQNQQQPSQVFPSLNLIPLTQLLTLGSDLQLLSPATGMAPGAQTIPLTLGSLNGQQQLQPQMLPIIVARLG
AQGTLLSSEELPLAPQIFTGLLIHPLFPGGILPSSQAGANPGVQNGVLPTGQAGANSAIQGTTQGHLPTPGVTDDDYGMSTPAGMRRGTHTPEETTTESPNGIH*
>AMTN Ictidomys (Spermophilus) tridecemlineatus
MKTTILLFCLLGSTQSLPKQFNLGLAPTKPAPDQASQLNQQQPNQVFSSLSLIPLTQLLTLGADLQLFNPAIGMAPGAQTLPLTLGALNGQQQLQSQMLPIIVAQLGAQ
GTILSSEELPLAPQIFTGLLIHPLFPGGIQPTNQAGANPDAQDGVLPAGQTGVNPTNQGTPASHLATPTGIDDDEFGETTPAGIRRGTQTTDETTTESPNRIH*
Lagomorpha
>AMTN Oryctolagus cuniculus
MKTAILLFCLLGSTQSLPKQLNPALELPPTKQAPDQETPLNQQQPTQVFPSLSLIPLTQMLTLLNPAAGMAPGAQTLPLTLEKLDGQQQLQPQILPIIVAQLGTQGTIL
SSEEVPAAPHIFTGLLLHPLFTSQTAANADSQDEVLPTGQAGVNPAIRATPADDDIGVTTPAGIQRSTPVTEETTSESPKGIQ*
>AMTN Ochotona princeps
MKTAILLFCLLGATQSLPKQLNPALGVPLPPTKQDRDQETPLKQQHPSQVFSSLSLIPLTQMLTLVSTLLLNPAAGAATGAQTLPLTMEILEAQQKLQTQMLPIIVAQL
GAQGAVLSSEELPAAPQILTGLLLHPMFTGKAGAHDDVQDEVLPTGKAEVSPATRGAQEGQFSTPVNDALGVTSPVGIQRGTPPTEETTTESPKGIH*
Laurasiatheria
Carnivora
>AMTN Felis catus
MKTMILLLYLLGSTQSLPTQLNPALGLPTKLALDQATLLNQQQLNQVFPSLSLIPLTQMITLGADLQLLNPPAGLAPGTQTLPLTLGGLNTQQPLQAQMLPVIVAHLGA
QGTILSSEELPMAPQIFTGLIFQPLFPGSTLPNSQANPDAQNGILPAGQAGMNPAIQGTSEGFSPTPSDTDDDFEVTAPTGIRRGMHTTQETTTGPPNGNQ*
>AMTN Panthera tigris
MKTMILLLYLLGSTQSLPTQLNPALGLPTKLALDQATLLNQHQLNQVFPSLSLIPLTQMITLGADLQLLNPPAGLAPGTQTLPLTLGGLNTQQPLQAQMLPVIVAHLGA
QGTILSSEELPMAPQIFTGLIFQPLFPGSILPNSQANPDAQNGILPAGQAE???AIQGTSEGFSPTPSDTDDDFEVTAPAGIRRGMHTTQETTTGLPNGNQ*
>AMTN Canis familiaris
MKTMILLLYLLGSTQSLPAQLNPALGLPPTKLTPHQATLLNQQQPNQVFPSLSLIPLTQMFTLASDLQLLNPAAGMASGTQTLPLSLGVLNTQQQLQPQMLPVIVAHLG
AHGAILSSEELPGSPQIFTGLIFQPLFPGAILPTSPANPDAQNGILPAGQAGGNPAIQGTPESFSTTPSDTDDDFGVTAPAGIQRGTHTTQETTSGPPNGTQ*
>AMTN Mustela putorius [JP005519]
MKTMILLLYLLGSTQSLPTQLNPVLGLPPTKPAPHQATLLNQQQPNQVLPSLSLIPLTQLFTLGSDLQLLNPAAGMPPGPQTLPLSLGVLNTQQQLQSQMLPVIVAHLG
AHGAIISSEELPVAPKIFTGLLFQPLFPGSILPTSPANPDAQNGILPAGQAGVNPAIQGTAEGFSTTPSDIDDDFGVTAPAGIQKGMHTTQETTTGSPNGIQ*
>AMTN Ailuropoda melanoleuca
MKTMILLLYLLGSTRSLPTQLNPALGLPPTKPAPHQAKLLNQQQPNQVFPSLSLIPLTQMFTLGSDLQLLNPAAGMAPGIQTLPLSLGSLNAQQQLQPQMLPVIVAHLG
THGAILSSEELPVAPQIFTGLLFQPLFPGAILPTSPANPDAQNGILPAGQAGANPAIQGTPEGFSPTPSDTDDDFGVTAPAGIQRGMHTTQETTAGPPNGIQ*
>AMTN Ursus maritimus
MKTLILLLYLLGSTRSLPTQLNPALGLPPTKPAPHQATLLNQQQPNQVFPSLSLIPLTQMFTLGSDLQLLNPAAGMAPGTQTLPLSLGGLNAQQQLQPQMLPVIVAHLG
THGAILSSEELPVAPQIFTGLLFQPLFPGAILPTSPANPDAQNGILPAGQAGASPAIQGTPEGFSPTPSDTDDDFGATAPAGIQRGVHTTQETTAGPPNGIQ*
>AMTN Odobenus rosmarus
MKTMILLLYLLGSTQSLPTQFNLALGLPPTKPAPHQATLLNQQQPNQVFPSLSLIPLTQMFTLGSDLQLLNPAAGMAPGTQTLPLNLGGLNTQQQLQPQMLPVIVAHLG
AHGAILSSEELPVAPQIFTGLLFQPLFPGAILPPSPANPDAQNGILPAGQAGVNPGIQGTPEGFSPTPSNTDDDFGVTAPAGIQRGMHTTQETSTEPPNGIQ*
>AMTN Leptonychotes weddellii
MKAMILLLYLLGSTQSLPTQLNPALGLPPTKPAPHQATLLNQQQPNQVFPSLSLIPLTQMFALGSDLQLLNPAAGMAPGTQTLPLNLRGLNTQQQLQPQMLPVIVAYLG
AHGAILSSEELPVAPQIFTGLLFQPLFPGAILPPSPANPDARNGILPAGQAGVNPAIQGTPEGFSPTPSDTDDDFGVTAPAGIQRGMHTTQETSTGPPNGIQ*
Cetartiodactyla

>AMTN Bos taurus
MKAAILLFCLLGSTLSLPMQLNPALVLPPTKLVPDQATLLNPQQPNQVFSSLSLIPLTHMLTLGSNLQLLNPAAGMPSGTQTLPLALGGLKVQQQLQPQMLPVIVAHFG
AQGTILSSEELQGTSQILTGLIFHPLFPGAILPTSQANPDAQNGILPAGQAGANPAAQGTPEDPFSTPSGTDDDFASTTPAGIQRGRPTTEETPTGSPKGIQ*
>AMTN Bos grunniens
MKAAILLFCLLGSTLSLPMQLNPALVLPPTKLVPDQATLLNPQQPNQVFSSLSLIPLTHMLTLGSNLQLLNPAAGMPSGTQTLPLALGGLKVQQQLQPQMLPVIVAHFG
AQGTILSSEELQGTSQILTGLIFHPLFPGAILPTSQANPDAQNGILPAGQAGANPAAQGTPEDPFSTPSGTDDDFASTTPAGIQRGRPTTEETPTGSPKGIQ*
>AMTN Bos indicus
MKAAILLFCLLGSTLSLPMQLNPALVLPPTKLVPDQATLLNPQQPSQVFSSLSLIPLTHMLTLGSNLQLLNPAAGMPSGTQTLPLALGGLKVQQQLQPQMLPVIVAHFG
AQGTILSSEELQGTSQILTGLIFHPLFPGAILPTSQANPDAQNGILPAGQAGANPAAQGTPEDPFSTPSGTEDDFASTTPAGIQRGRPTTEETPTGSPKGIQ*
>AMTN Pantholops hodgsonii
MKAAILLFCLLGSTLSLPMQLNPALVLPPTKLVPDQATLLNPQQPSQVFSSLSLIPLTHMLTLGSDLQLLNPAAGMPSGTQTLPLALGGLKVQQQLQPQMLPVIVAHFG
AQGTILSSEELQGTSQILTGLIFHPLFPGAILPTSQANPDAQNGILPAGQAGANPATQGTSEDPFSTPSGTDDDFAATTPAGIQRGRQTTEETPTGSPKGIQ*
>AMTN Capra hircus
MKAAILLFCLLGSTLSLPMQLNPALVLPPTKLVPDQATLLNPQQPSQVFSSLSLIPLTHMLTLGSDLQLLNPAAGMPSGTQTLPLALGGLKVQQQLQPQMLPVIVAHFG
AQGTILSSEELQGTSQILTGLIFHPLFPGAILPTSQANPDAQNGILPAGQAGANPATQGTPEDPFSTPSGTDDDFAATTPAGIQRGRQTTEETPTGSPKGIQ*
>AMTN Ovis aries
MKAAILLFCLLGSTLSLPMQLNPALVLPPTKLVPDQATLLNPQQPSQVFSSLSLIPLTHMLTLGSDLQLLNPAAGMPSGTQTLPLALGGLKVQQQLQQVSSKPYSMLPV
IVAHFGAQGTILSSEELQGTSQILTGLIFHPLFPGAILPTSQANPDAQNGILPAGQAGANPATQGTPEDPFSTPSGTDDDFAATTPAGIQRGRQTTEEAPTGSPKGIQ*
>AMTN Sus scrofa
MKTTILLFCLLGSTLSLPMQFNPVLGLPPTKLVPDQATLRNQQQPNQVFPSLSLIPLTQMLTLGSDLQLLNPAIGMVPSSQTLPLTLGALNVQQQLQPQMIPVIVAHLG
AQGAILSSEELPATRQILTGLIFHTLFPGAILPPSPAKPDAQNGIHPAGQAGANPAVQGTPRGPFPTSSGTDDDFDVTTPAGLQRGTHATEETTTGSPNGMQ*
>AMTN Vicugna pacos
MKTMILLFCLLGSTLSLPMQVNPTLGLPPAKLVPDQATPLNQQQPNPVFPSLSLIPLTQMLTLGSDLQLLNPGMAPGSQTLPLTLGGLNVKQQLRPQILPVIVAHLGVQ
GTILSSEELPAAPQIFTGLIFHPLFPGAIQPTSQANPDVQNGILPAGQAGVNSANQGTPRGPLPTSSGTDDDFVVTTPAGIQRGTHTMEETTTPSPNGIQ*
>AMTN Camelus ferus
MKTTILLFCLLGSTLSLPMQLNPTLGLPPAKLVPDQATPLNQQQPNPVFPSLSLIPLTQMLTLGSDLQLLNPGVAPGSQTLPLTLGGLNMKQQLQPQILPVIVAHLGVQ
GTILSSEELPAAPQIFTGLIFHPLFPGAIQPTSQANPDVQNGILPAGQAGVNSANQGTPWGPLPTPSGTDDDFVVTTPAGIQRGTHTMEETTTPSPNGIQ*
>AMTN Orcinus orca
MKATILLFCLLGSTLSLPMQLNPALVVPPTKLVPGQATLLNQQQPNQVFPSLSLIPLTQMLTLGSDLQLLNPAAGMAPGTQTLPLTLGGLKVQQQLQPQVLPVIVAHLG
AQGAILSSEELPATPQILTGFIFHPLFPGAILPTSQATPDVQNGILPAGQAGANPAIRGNSEGPFPTTSGTDDDFAETTPVGIQRGIHTTEETTTGSPKGI*
>AMTN Tursiops truncatus
MKATILLFCLLGSTLSLPMQLNPALVVPPTKLVPGQATLLNQQQPNQVFPSLSLIPLTQMLTLGSDLQLLNPAAGMAPGTQTLPLTLGGLKVQQQLQPQVLPVIVAHLG
AQGAILSSEELPATPQILTGFIFHPLFPGAILPTCPPAHLPTCPPDVQNGILPAGQAGANPAIRGNSEGPFPTTSGTDDDFAETTPVGIQRGIQTTEETTTGSPKGI*
>AMTN Physeter catodon
MKTMILLFCLLGSTLSLPMQLNPALVLPPTKLVPDQATRLNQQQPNQAFPSLSLIPLTQMLTLGSDLQLLNPAAGMAPGSQTLPLTLGGLKVQQQLQPQVLPVIVAHLG
AQGTILSSEELPATPQILTGFIFHPLFPGAILPTSQATPDVQNGILPAGQAGANPAIQGTPEGPFPTTSGRDDNFAETTPAGLQRGTHTTEETTTGSPKGI*
>AMTN Balaenoptera acutorostrata
MKTTILLFCLLGSTLSLPMQLNPALVLPPTKLVPDQATLLNQQQPNQVFPSLSLIPLTQMLTLGSDLLNPVAGMAPGTQTLPLTLGGLKVQQQLQPQVLPVIVAHLGAQ
GAILSSDELPATPQILTEFIFLPLFPGAILPTSQATPDVQNGILPAGQAGANPAIQGTPEGPFPTTSGTDDDFAETTPAGIQRGTHTTEETTTGSPKGI*

Eulipotyphla

>AMTN Erinaceus europaeus
MKTTFLLFCLLGSTHTLPMQLNPALGLPPSKLVPDQATLLSQPQPNQVFPSLNLIPLTQMLTLGSDLQLLNPTAGMTPGPQTLPLTLGGLNVQQQLQPQMLPIIVAQLG
AQGTILSSEELPVASQILGLLFQPLLPGSILSTSKANPDTQNGILPSGQVGANPAIQEDTEAPFSTPSGIDDDFGMTTPAGIQRGLQPTEETTTGSPNGLQ*
>AMTN Sorex araneus
MKTTILLLCLLGSTQSLPMQLNHALGLPPINLVPDQATLNQQQSNQVFPSLSLIPLTQILTLGSDLQLRNAPAPGAQTLPLSLGAVNGQQQLQPQMLPVIVAQLGAQGT
ILSSEELPVASPLLTGLVFQPLLQGAILPNSQINPDLHNRILPAGQAKPNPALHGTPEGPLPTPSGIDDDFVVTTPAGIPRGLHLPEETTTESPEGFQ*
>AMTN Condylura cristata
MKTTILLFCLLGSTQSLPMQLNPVLGLPPTKLVPDQATLLNQQQPTQVFPSLSLIPLTQMLTLGSDLHLLHPTAGITPGTQTLPLTLGGLNAQQQLQPQMLPVIVAQLG
AQGTILSSEELPVSQHIFAGLIFQPLFPGAILPTSQANPDAQNGILPAGQVGINPAIQGTPEGPFLTPSAIDDDYGVTTPAGIQRGLHTTEETTTGLPDGIQ*

Chiroptera

>AMTN Eptesicus fuscus
MKTVILLLYLLGSTQSLPMQFKPALGLPPTQLVPDQTTRLNQQQPNQVFPSLSLIPLTPMLTMGSDLQLLNPPPARMAPAAHTLPLTLGGLNVQQQLQPQMLPLIVAQL
GAQGTILSSEELPVAPQIFTGLIFHPLFSGAILPTSHANPDVQNTILPAGQTGVNPAIQGTPEGPSPTPSGTDDDFEVTTPAGIQRGMHIAEETTTEFPNGIQ*
>AMTN Myotis davidii
MKTVILLFYLLGSTQSLPMQLKPALGLPPTQLVPDQTTLLNRQQPHQVFPSLSLIPLTQMLTMVSDLQLLNPPPAGMAPAAHTLPLALGGLNVQQQLQHQVLPLIVAQL
GAQGTILSSEELPVAPQIFTGLIFHPLLSGAILPTSQANPDIQNTILPAGQAGVNPAIQGAPEGPSPTPSVTDDDFEVTTPAGIQRGMHIAEETTTGFHHGIQ*
>AMTN Myotis lucifugus
MKTVILLFYLLGSTQSLPMQLKPALGLPPTQLVPDQTTLLNRQQPHQVFPSLSLIPLTQMLTMVSDLQLLNPPPAGMAPAAHTLPLALGGLNVQQQLQPQMLPLIVAQL
GAQGTILSSEELPVAPQIFTGLLFHPLFSGAILPTSHANPDIQNTILPAGQAGVNPAVQGAPEGPSPTPSGTDDDFEVTTPAGIQRGMHIAEETTTGFPHGIQ*
>AMTN Pteropus alecto
MKTTILLFCLLGSTQALPIQLNPALRLPPTKLVPDQATLLNQQQPNQVFPSLGVIPLTQMLTLGSDLQLLNPAAGMAPAAQTLPLTLVALNVQQQLQPQMLPVIVAQLG
AQGTILSSEELPVAPQIFAGLIFQPLLSGAILPTSQANPDVQNGILPAGQAGINPPIRRAPGSPFPTPSGAGNDFGVTTPAGIQRGMLTTEETTTGSPSGIR*
>AMTN Pteropus vampyrus
MKTTILLFCLLGSTQALPIQLNPALRLPPTKLVPDQATLLNQQQPNQVFPSLGVIPLTQMLTLGSDLQLLNPAAGMAPAAQTLPLTLGALNVQQQLQPQMLPVIVAQLG
AQGTILSSEELPVAPQIFAGLIFQPLLSGAILPTSQANPDVQNGILPAGQAGINPPIRGAPGSPFPTPSGAGNDFGVTTPAGIQRGMLTTGETTTGSPSGIR*

Perissodactyla

>AMTN Equus caballus
MKTVILLFCLLGSTLSLPMQFPALGLPPTKPVPDQATQLNQQQPNQVFPSLNLIPLTQMLKLGSDLQLLNPAAGVAPGAQTLPLTLGGLKAQQQLQSQMLPIFVAQFGA
QGTILSSEELPAAPQIFAGLIFQPLFPGGILPTSQATPDVQNGILPAGQGGVKPAIQGTSESPLPTTSDTDDDFGGTTPAGIQRGMRTTEETITKSPNGIQ*
>AMTN Ceratotherium simum
MKTAILLFCLLGSTLSLPMQLSPPLGLPPTKLVPDQATLLNQQQPNQVFPSLSLMPLTQMLTLGSDRQQLNPAAGVAPGAQTLPLTLGGLKAQQQLQSQMLPVFVAQIG
AQGTILSSEELPVARQIFTSLIFQPLFPGAILPTSQANPDVQNGILPAGQGGVKPAIQGTSESPFPTPSDPDDDLGGTTPAGIQRGTHTTKETTTRSPNGIQ*

Xenarthra

>AMTN Dasypus novemcinctus [JX523656] (pseudogene)
??????????????????KQFNSALALPSAKLVPGQETLLNQQLPNQVFPLTLIPLTQMLTLGSDLQLLNPSAEITSSTQTIALTLGDLYKQQQLQPQILPIILAQLGA
QGIILSSEELPVAPQIFTGFLIQSLFPGAILPTTQPEANPDVQCGVLPAGQAGINPSIQGTPEGYFPIPRAERMALE*PFLEASKGTHTPLRKPP*SYQVEFIK

>AMTN Choloepus hoffmanni [JX523657] (pseudogene)
??LFSSLSLVPLTQMLAVESDLQLVNLSARMVTGVQTLPLTQGT*TYSSSCSHKCYQLL*HNLES
RVQS*TQRNCQRLHKSSQPSLSSSQEPSCPPVRQELIQMCSMESFLPDKQE*ILPSWEHQKTTSQNPVAQKMDLE*PPLQISKGADTPPRKPPQSHQMGLKKLFK
Afrotheria
>AMTN Loxodonta africana
MKTTILLFCLLGSTQSLPQLNPALGLSAAKLVPDQATLLNQQQPNQVFPSLSLIPLTQLLTLGSDLQQLNPATGMAPGTQTFPLNLGGLTIKQQLQSQLLPIIVAQLGA
QGAILSSEELPMAPQIFAGLLIQPLFPGAILPTSLAGATPEVQEGILPAGQAGLNPAIQRTPEKHPSTSSDTDSVFGVTTPAGLQRGMRTTGETTTESPNEIQ*
>AMTN Procavia capensis
MKTMILLFCLLGSTKSFPKQPNPALGFPPAKPVPDQATPLNQQQVSVLPSLSVIPLTQMFTLGSDPQLINPAAGTAAGAQSLPVNLGQLTLQQLQ??????????????
?GAILSSEELPTTPQIFTGLLIQPLFPGAILPTSQAGVTPEVPEGALPAGQAGVNPAIQGTPEGRLPAPSAMDDMSGVTSPAGLQRSIYTTEETTTDSPDGIQ*
>AMTN Trichetus manatus
MKTMILLFCLLGSTQSLPSQLNTALGPLPAKLVPDQATLLNQQQPDQVFPSLSLIPLTQLLTPGSDLQLLNTATGMAPGAQTLPLNLGGLAVQQQLQSQLLPIIIAQLG
AQGVILSSEELAVAPQIFAGLLIQPLFPGAILPTSQAGATPEVQEGILPAGQAGVNPAIQRTPEGHLPTPSGTDDMYGVTTPSGLQRGMHTTEETTTDSPDGIQ*
>AMTN Echinops telfairi
MKTIILLFCLLGSTQSLPPLNPALGLPPVKLVPEQSTLLNPQQPNQVFPSLSLIPLTQMLALGSDLQMLNTATGVGPGTQTLPVNLGGVTVQQQLQPQMLPIFVAQLGT
QGAILSSEELPVAPQVYTGLLIHPWFPGAFLPTSQAGSTPEGQDGALPAGQAAAHPAIQGTPEGHLPTPSGTDYLSGVTAPAGLQRDMHTTEEATTDGTQ*
>AMTN Chrysochloris asiatica
MKTMILLFCLLRSTQSLPMQPNPALGLPPAKLVHDQATQLNHQQPNQVFPSLSLIPLTQMFTLGSDLQLLNPASGMAPGAQTHPLNLGGLTVQQQLHPQMLPVIVAQLG
AQGAILSSEELPVAPQMFTGLLIHPLFPGVMLPTSQAGPTPEIHEGILPAGQAGVNSAIQGTLEGHLPTPSGTDYMMGVTAPAGLQRGTYTTEETTTESPNGIQ*
>AMTN Elephantulus edwardii
MKTIILLLFLLESTQSLPIQLNPALGLPQAKLVPDQTTLLNQQQPNQVFTSLGLIPLTQLLTLGSDLHLLNPSTGLAAGAQTLPLNLGALTVQQQLQSQMLPIIVAQLG
AQGVILSSEELPVAPQIFAGLLIQPLYPGAILPTSQAGATPELQEGTLPAGQTGVNPAIQRTPEDHLPTPTGTDDVFGGTTPASLQRSMYTTEETTTESPDGI*
>AMTN Orycteropus afer (pseudogene)
???MLPIIVAQLG
DQGTIINSEELPVAPQIFTGLLIQPLFQEPPCLPIRQGLLQRSRTESFPQNKQE*IMPSSKPQKVTSQLPVAQMICLE*PLLLTSKGTCTPLRKLPLNHQMRHTE
Marsupiala
>AMTN Monodelphis domestica
MKTAVLLFCLLGTIQSLPILQLMSMYPVPGHGQQQAQQLYPGVGLPPPKLGLEQPALFTPQQSNQLFPPLGQIPLTQMFSVGTDMQLMTPATGLLPGIQILPMTLGDTS
IAPQVNPQQMLPVLVAQIGAQGAVRSSEELPIAPQIFTGLLIQPFGTGATLPTGQSGIDANTQDAALPAGQTGGNPAIWGTSEGQLPTPSSPDDVFEATIPVGIQKATE
GSTTEAPNGTD*
>AMTN Sarcophilus harrisii
MKTAVLLFCLLGTIQSLPILQLMRMYPVQGFGQQQVQQQLHSGVGLPPTNLVLDQPTLLTPQQPSQLFPPLSQIPLSQIFSVGTDMPLINPATGLVPNIQILPMTLADT
NIAQQVSPQQVLPVIVAQIGAPGAFLSSEELPMAPQIFTGLLIQPFGTGAILPTGQERIDANTQDAALPAGQTGGNSAIWGTPEGQLPTPSGPDDVFEATNPVGIQRIE
GSTTEAPNGTD*
>AMTN Macropus eugenii
MKTAVLLFGLLGTIQAFPILQLMRDVSSTRPWSTASTGIQFVPGMGLPPANLVLDQSALLTPQQPNQ??????????????????????INPAAGLVPSIQIIPMTLTD
ANTPQQVSPQVLPVIVAQIGTQ?????????PMAPQIFTGLLIQPFGTGAMLPTGQEKINANAQDTALPAGQTGGNPIFWGAPEGQFPTPSGPDDVFEATIPDGIQRTA
EGSTTEAPNGTD*
Monotremata
>AMTN Ornithorhynchus anatinus
MKTTILLLCLLGTTRSLPMLQILRMASFRDNIPQQIQQVNPAFGFLPAQLVVDHSILQTQQDSNQMIPLIVAQVGAQGAILSSEELIFTGLFIQPLAPGRMAPIGQDNP
EDQDQVLPTGQSGMSPATQDPPEDQPPTASGLLDESEVTLPANLQGVSLTTEESTTEEPRGNQ*

SAUROPSIDA
Crocodilia
>AMTN Alligator mississipiensis
MKTVILLLLCLLGTAFSLPVRQAGTLPVSNSREILQLMRLYSSLGNIPQQTQQQVNPGLGLPPAQLLPDQTALLAQQANQVFPTLNFIPLTRVWPFGADINLVNGAAGGV
PITQTLPTGAGGANTQHLVPQQGVLPLILGQMGPQGLGVSSEEMQTDAQILAGLILLGLQGLLQANQAVANSEGLDIVFPAGQTGVNQGSPGQLPSPEGTADMSGATLP
AGIKKGSQTPESITEAASGVYTTPPGFRQLDSEQVTDAIFAEPAILLNMEANEIQAPPTTDIGTGTVHSINLHTQGYDNKIGVSPVRGDRALPLANIDVKALREYKSRS
A*
>AMTN Alligator sinensis
MKTVILLFCLLGTAFSLPVRQAGTLPVSNSREILQLMRLYSSLGNIPQQTQQQVNTGLGLPPAQLLPDQTALLAQQANQVFPTLNFIPLTRVWPFGADINLVNGAAGGV
PITQTLPTGAGGANTQHLVPQQGVLPLILGQMGPQGLGVSSEEMQTDAQILAGLILLGLQGLFQANQAVVNSEGPDIVFPAGQTGVNQGSPGQLPSPEGTADMSGATLP
AGIKKGSQTSESITEAASGVYTTPPGFRQLDSEQVTDAIFAEPAILLNMEANEIQAPPTTDIGTGTIHSINLHMQGYDNKIGVSPVRGDRALPLANIDVKALREYKSRS
A*
>AMTN Caiman crocodilus [KM069444]
MKTVILLLCLLGTAFSLPVRQAGTFLPVSNSREILQLMRLYSSLGNIPQQTQQQVNPGLGLPPAQLLPDQTALLAQQANQVFPTLNFIPLTRVWPFGADINLVNGAAGG
VPITQTLPTGAGGANTPHLVPQQGVLPLILGQMGPQGLGVSSEEMQTDAQILAGLILLGLQGFLQANQASINSEGPDIVFPAGQTGMNQGAPGQLPSPEGTADVSGATL
PAGIKGSQTPESITEAASGVYTTPPGFRQLDSEQVTDASFAEPAILLNMEANEIQAPPTTDIGTGTINSINLQTQGYVNQIGVSPVRGDRALPLANRDVRALREYKSRS
A*
Squamata
>AMTN Ophiophagus hannah
MEVIILLLSLLGLTFSTPINHIGGSLATSSSKEVLRLLHRYIAISNTPQQAQSRQRAILPPAKLVPDLNLPGNQPPNVNLPVLSVPQNQIFPIDMNYLSTLLGILSTIQ
TLPIVGGGMTVPQQLPPGQAMLPIIVTQMGPQGAVLSSEEMSPSQFFTGLLIPGMQGAMFPSGQAEPPPEAQDGFLPVGQANPEGTAAGIQKVFPTATDGLSEIPGELY
PTPSGFRQSDTGTSGLFLEPTADINMEPSELREPPTSPVKFDMSNMQSPHNLSQPLVRGDSHIPMNTVASKPLNLP*
>AMTN Python molurus
MKIVILLLCLLGLAFSKPVNQAARILATSNSREILRLLQRYRATGNTPQQTQPRPRVVPGLPPAKLVPDMNLPENQLPNGVTPFEWPFTNFPVISQLAEVGGPNLQNFP
VFSIPQNQMYPIDWNYLTTLLGILSTMQALPAAGGGMTIPQQLLPGQPMLPIIVAQMGPQGAVLSSEEMSPSQFFTGLLIPGMQGALLPSGQAEPPPEGGEELLPAGQA
GSNPEGTAAGIQKVFPTTTDGLSEIPDELYPTPSGFRQPDVGTSGMFIQPTVDISMEPSELREPPTSLVKLDMGNMQSPHNLSQPLVRGDSHILVNTVASKPLNVP*
>AMTN Python regius [KM069438]
MKIVILLLSLLGLAFSKPVNQAARILATSNSREILRLLQRYRATGNTPQQTQPRPRVVPGLPPAKLVPDLNLPENQLPNGVTPFEWPFTNFPVISQLAEVGGPNLQNFP
VFSIPQNQMYPIDWNYFTSLLGILSTMQALPAAGGGMTIPQQLLPGQPMLPIIVAQMGPQGAVLSSEEMSPSQFFTGLLIPGMQGALLPSGQAEPPPEGGEELLPAGQA
GSNPEGTAAGIQKVFPTTTDGLSEIPVELYPTPSGFRQPDVGTSGMFVQPTVDISMEPSELREPPTSLVKLDMGNMQSPHNLSQPLVRGDSHIPVNTVARKPLNVP*
>AMTN Crotaphytus insularis (partial) [KM069443]

?????????????????????????????????????VQRYKAQENSPQQTQQRPNSGVGLPPAKLVPDQQPLANQIPNEILPFEWPFANFPIISPQNPLELGNPNVQN
LPGFSVLPLTQMVPIDINSLAALLGVLSATQIQPLPGGGITGPQQLLPAQSMLPIIFTQMGPQGTVLSSEEMPMSQVFAGFLVPGMQGGLLPSGQAGTIPEGQEEPLPA
GQAGTNQGNLPYPESTAAGIQKGSPTTGDGLREATSISYPSPSGFRQPGVVTNEVFIEPTVGINMEPSELRETT???????????
>AMTN Crotalus mitchellii
MKIIILLLSLLGLTFSIPINHVGRILATSSSKEILRLLQRYIAVGNTPQQAQSRQRPVLPPAKLVPDPSLPGNQPPNVNFPVFSLPQNQVFPIDMTYLSNLLQILSTMQ
TLPIFGGGMTVPQQLPPGQAMLPIIVTQMGPQGAVLSSEEMSPSQFFTGLLLPGMQGAMFPSGQAEPPPEGQDGLLPVGQANPEGTAAGIQKVFPTATDGLSEIPGELY
PTPSGFRQPDAGTSDMFVEPTADINMEPSELREPPTSLVKFDMGNMQSPHNLSQSPVRGDSPIPMNTVASKPLNVP*
>AMTN Anolis carolinensis [KM069435]
MKIVILLLSLLGLTFCLPVNQFGRRFATSNSREILRLMQRYKAQGNVPQQTQQRSNPGIGLPPAKLVPDQHPLANHIPSEIPAFERPFANYPILTAQNPQELGSPTLQN
FPGFNVLPLTQMVPIDINYLALLLGALSTTQTQPLPGGGLNIPQQLLPPPQSMFPIIFTQMGPQGTVLSSEEMPTPQVMAAFLLPGLPGGLFPSGQSGALPEDQDELLP
AGQVGTNQGNLPFPESTAAGIQKGSPATEDGLNVAPGAFYPTPSGFRQPGVVTNEVFVEPTGGIHMEPSELREPPTSLVGLDKGNNQKLQNLSQSPVRGDSYMPMTTTA
SKPLKAP*
>AMTN Timon tangitanus (partial) [KM069439]
???????????????????????????????????RDVQRYKAMGNNPQQTQQRPRLGPGLPPAKLVPDQNPSVNQVPNEQVAPLEWTFANFPVTSNFPQNPSELGGPN
MQNFPGYNVLPFPMDLNSLAILLGALTLPQTLPVAGVGTTMPQQLLPPQSMLPIIVAQMGPQGAVLSSEEMPPQSQIVGVLVPGGGFFPLGQAGALPEGQEGLLPANQA
GSNQGNLPFPDSTAAAIQKISPTASDGLSEVPSGLYPTPSGFRQPGPVTNGVFAEPTAGMNMEPSELQGTTK????????????
>AMTN Takydromus sexlineatus (partial)[KM069441]
????ILLLPLLGLTISLPLNQMGRILATSNSREILRLMQRYKAMGNNPQQTQQRPRLGAGLPPAKLVPDQNPSVNQVPNEQAVPVEWTFANFPVTSNFPQNPSELGGPS
IQNIPGYNVLPFTQMLPMDLNTLAILLGALTLPQTLPGAGVGTTMPQQLLPPQPMLPIIVAQMGPQGAVLSSEEMSPQSQIVGVLIPGGGFFPLGQAGALPEGQESLLP
ANQAGSNQGNLPFPEGTAAAIQKISPTASDGLSEVASGV??????
>AMTN Tarentola mauritanica [KM069446]
MKIVILFLSFLGLTIALPISPLERAIATSNSREILRLMQRYRAMGNNPQQTQQRSNPGFGLPPAKLVPDQTALANQLPNEVTPLEWPFTNFPMTPPKIPSELGTTNLQH
IPGFNLLPFTQMIPLDINTLTTLLGLGGLPSTQTLPVGGVNVPQQLLPPSQILPIIFAQMGPQGAVLSSEEAQSPSQFFTGLILPGMQGILIPSGQAEALPEGQEGLLP
AGQAGSNPANSAGQLPFPEGTADATAPAGIQKVSPGMHDDLSGTANGLNPTPSGFRQPGYDHGATQDPFAERTIFVKLNMEPSELREPPTTMARPEDDNSLVGHTLAQS
LVRGDRHMPVTTVVSKLLREP*

AMPHIBIA
Anura
>AMTN Xenopus (Silurana) tropicalis [KM069437]
MVMFLLLCLLRASLGFPMLHLMQMFNLLGRVPEQTQRIPHLFVAPSQLVQLHGTQLRYMHPFQMSPIMFAQLRQQALSGSDSEENVLPGLIMIPLSAGLDENIATVHQA
GVIIDNSILQEQPVLNPAGQHGVSTAIQNKILPELPWVIPTQAAQNDVSVTGIIKGSYTDPLPTNTPDIDLNAPEPTMSMGRDNDLTVNAHTMGEENWILCNSYVPDLC
ELHNIVRGDGYIPIKTPVYFKNGQIQGNRNTESY*
Caudata
>AMTN Pleurodeles waltl [KM069436]
MRTTILLLCLLGTSIAFPVREAERILSASNSGEMLQLMQLYNMLGNTPEQTQQVHPGHGLPPAQLIPDMTAQQTQQQLSQFQPSENVFGNLPILPPLSMPPGFPQSPGN
QPPGVMPYPQALPMNVGEMTVHHSMFPHPHHLFPLIITQMGQQGLLMSSEEMPFLPQVFAGVIMPPFGPILPALPVLPGVPAFAGLPGLPPLLQGAARPAAGAGISLDE
QEAIPMGQAGLNPNKQRETASPAPGVTPAQDTGEVLAVTVPAGLRKGVTGTDSAGLLQTFTPPAGLRQFPTESATEVMFVEPTGLVTETSPDGVDERLPHSYRPQNQRF
NSVARGDQNLAMAVPDLKPLRKHKKRRA*

COELACANTHIFORMES
>AMTN Latimeria chalumnae
MKTIMLLFCFLGTAVAVPVHSGVRSVHGGVAASNSRERTPLLVSQQPQLQQGQPRILQLQTFPERVQVPVQPNIQGQQQLLPSYRFVPLTQLLAQGPGVHAQQALAVNI
PQLVPQSTSSEEMVQMSTGVLIPIVVDPAVPGVPAIEMPVPGGILPNPGGNPSTQGGDTPIPGNPVGGQPTVPTGVRKIAPTAEVSVTDAVNQGIFQPVAGATPTAAGL
KKTVQDEDRPDVIFVEPSPPCDTIPNGMKQLTFSSELITVSPAEASITISSVTNSMPKNKWHIASKAGLRGDIAGSLKAAGKKPIQNY*
