# Supplementary material for: Amelotin: an enamel matrix protein that experienced distinct evolutionary histories in amphibians, sauropsids and mammals
Source: BMC Evol Biol. 2015 Mar 14;15:47. doi: 10.1186/s12862-015-0329-x (PMC4373244; doi:10.1186/s12862-015-0329-x)
Supplement: Additional file 3: — Forward (F) and reverse (R) primers used to amplify Amelotin cDNA of various species and to build AMTN probes. Target regions and annealing temperatures are indicated. [file 12862_2015_329_MOESM3_ESM.pdf]

Species	Primers	Target region	Annealing temperature
Anolis carolinensis	F: CAACAGCAAGATACCAATTTGG<br>R: GCAGTGACACACATCATTGG<br>Probe:<br>F: GGTTCCAGATCAGCATCCAT<br>R: ATTCAGGCCATCTTCAGTCG	5' UTR to 3' UTR<br><br>exon 3a to exon 8	50°C<br><br>58°C
Python regius	F: AGTCGTGGGGTAAGAGATGC<br>R: TGCTTTCCCTTTCTGAGCTG	5' UTR to 3' UTR	59°C
Takydromus sexlineatus	F: ATGAAGATTGTAATTTTACT<br>R: CATTTCCTCAGAGCTGAGCA<br>F: ATATTACGGCTGWTGCAAAG<br>R: GGTGGTTCCCGAAGTTCACTTG	exon 2a to exon 7<br><br>exon 2c to exon 8 (3' end)	50°C<br><br>50°C
Tarentola mauritanica	F: CTACAAGCAACAGCAGAGA<br>R: CTRTCTCCTCTCACARGTG	exon 2b to exon 8 (3' end)	50°C
Caiman crocodilus	F: TTTACCAGTGCGACAAGCTG<br>R: TCTCCTCTCACAGGGGATACAC	exon 2b to exon 8 (3' end)	60°C
Xenopus tropicalis	F: GTTCCACAGAAATATATGGTCATG<br>R: GCAACTCTAAACAAATCTACAGAC	5' UTR to 3' UTR	55°C
Pleurodeles waltl	F: AGAAACATTCGGGACTGGTG<br>R: TTGCCCAGATTGTGATCGT<br>Probe:<br>F: ACCAGGCTTTCCACAGTCAC<br>R: ATCACCTCTCGCTACGCTGT	5' UTR to 3' UTR<br><br>exon 3b to exon 8 (3' end)	60°C<br><br>62°C

Additional file 3
